# *Gata2*-L359V impairs primitive and definitive hematopoiesis and blocks cell differentiation in murine chronic myelogenous leukemia model

**DOI:** 10.1038/s41419-021-03826-1

**Published:** 2021-06-02

**Authors:** Ya-Kai Fu, Yun Tan, Bo Wu, Yu-Ting Dai, Xiao-Guang Xu, Meng-Meng Pan, Zhi-Wei Chen, Niu Qiao, Jing Wu, Lu Jiang, Jing Lu, Bing Chen, Avigail Rein, Shai Izraeli, Xiao-Jian Sun, Jin-Yan Huang, Qiu-Hua Huang, Zhu Chen, Sai-Juan Chen

**Affiliations:** 1 Shanghai Institute of Hematology, State Key Laboratory of Medical Genomics, National Research Center for Translational Medicine, Ruijin Hospital Affiliated to Shanghai Jiao Tong University (SJTU) School of Medicine, Shanghai, China; 2 Institute of Health Sciences, Shanghai Institutes for Biological Sciences and Graduate School, Chinese Academy of Sciences and SJTU School of Medicine, Shanghai, China; 3 Cancer Research Center, Sheba Medical Center, Sackler Faculty of Medicine, Tel Aviv University, Tel Aviv, Israel; 4 Division of Pediatric Hemato-Oncology, Schneider Children’s Medical Center of Israel, Sackler Faculty of Medicine, Tel Aviv University, Tel Aviv, Israel; 5 Present Address: Department of Rheumatology, Renji Hospital Affiliated to Shanghai Jiao Tong University School of Medicine, Shanghai, China

**Keywords:** Haematopoiesis, Chronic myeloid leukaemia

## Abstract

GATA2, a key transcription factor in hematopoiesis, is frequently mutated in hematopoietic malignancies. How the GATA2 mutants contribute to hematopoiesis and malignant transformation remains largely unexplored. Here, we report that *Gata2-*L359V mutation impeded hematopoietic differentiation in murine embryonic and adult hematopoiesis and blocked murine chronic myeloid leukemia (CML) cell differentiation. We established a *Gata2-*L359V knockin mouse model in which the homozygous *Gata2-*L359V mutation caused major defects in primitive erythropoiesis with an accumulation of erythroid precursors and severe anemia, leading to embryonic lethality around E11.5. During adult life, the *Gata2-*L359V heterozygous mice exhibited a notable decrease in bone marrow (BM) recovery under stress induction with cytotoxic drug 5-fluorouracil. Using RNA sequencing, it was revealed that homozygous *Gata2-*L359V suppressed genes related to embryonic hematopoiesis in yolk sac, while heterozygous *Gata2-*L359V dysregulated genes related to cell cycle and proliferation in BM Lin^-^Sca1^+^c-kit^+^ cells. Furthermore, through chromatin immunoprecipitation sequencing and transactivation experiments, we found that this mutation enhanced the DNA-binding capacity and transcriptional activities of Gata2, which was likely associated with the altered expression of some essential genes during embryonic and adult hematopoiesis. In mice model harboring *BCR/ABL*, single-cell RNA-sequencing demonstrated that *Gata2-*L359V induced additional gene expression profile abnormalities and partially affected cell differentiation at the early stage of myelomonocytic lineage, evidenced by the increase of granulocyte–monocyte progenitors and monocytosis. Taken together, our study unveiled that *Gata2-*L359V mutation induces defective hematopoietic development and blocks the differentiation of CML cells.

## Introduction

Hematopoietic differentiation is orchestrated by precise transcription programs and epigenetic regulation in distinct stages, and the dysregulation of key transcription and/or epigenetic factors may induce hematopoietic failure or malignant transformation^1,2^. High-throughput sequencing can be useful to identify malignancy-related mutations in these regulators^3^. However, the function of mutated regulators in normal and malignant hematopoiesis remains unexplored in many instances.

GATA2, a key transcription factor determining the differentiation/self-renewal fate of hematopoietic stem cells (HSCs), also acts as one of the core regulators in erythrocytic differentiation^4,5^. The complete knockout (KO) of murine *Gata2* results in hematopoietic failure and embryonic lethality^6,7^. Embryonic stem cells lacking *Gata2* fail to undergo definitive hematopoiesis and exhibit defects in the production of all hematopoietic lineages^6,8,9^. Deleting the enhancer of *Gata2* also impairs the self-renewal of HSC and leads to impeded differentiation of erythropoietic progenitors in murine embryos, indicative of sufficient expression of *Gata2* being required for the embryonic erythropoiesis^10^. Intriguingly, the ectopic overexpression of *Gata2* reduces the expression of cell cycle-related genes, such as *CDK4* and *CDK6*, facilitating the quiescence of HSCs^11,12^. Maintaining the accurate expression and function of *Gata2* is thus pivotal for normal hematopoietic differentiation.

*GATA2* is frequently mutated in hematopoietic malignancies, including acute erythroid leukemia^13,14^, acute myeloid leukemia (AML), and myelodysplastic syndrome (MDS)^15,16^. Aberrant GATA2 expression is associated with poor clinical outcomes in MDS and AML^17,18^. Most *GATA2* mutations occur in the GATA2 DNA-binding domains such as the zinc-finger domains 1 (ZF1) and 2 (ZF2), and these mutations are believed to affect the transcriptional function of GATA2^19^. By comparing the DNA-binding affinity with wild-type (WT) GATA2, *GATA2* mutations are generally classified as “loss-of-function” (such as A318T, G320V, and T358N) or “gain-of-function” ones (such as L359V)^20^. Both functional forms of mutations can be found in hematopoietic malignancies, assumedly disrupting the hematopoietic differentiation of HSC and progenitor cells^21^, but the in vivo functions of these mutations are rarely reported and need further study.

*GATA2-*L359V mutation, initially identified in patients with myelomonocytic transformation of chronic myelogenous leukemia (CML)^22^, also exists in patients with AML and is associated with poor prognosis^16,23^. The preliminary in vitro study showed that this mutation could not only increase the DNA-binding capacity and the transcriptional activity of GATA2 but also enhance its inhibitory effect on PU.1, an important regulator of myelopoiesis^16,21,22^. However, the functions of such mutations in vivo, particularly the underlying regulatory mechanism in hematopoiesis and malignant transformation, remain largely unknown.

In the current study, we established a *Gata2-*L359V mutation knockin mouse model, intending to examine the effect of this mutation on the murine hematopoiesis and the differentiation block of CML cells.

## Results

### Homozygous *Gata2-*L359V mutation is associated with embryonic lethality

**Fig. 1:**
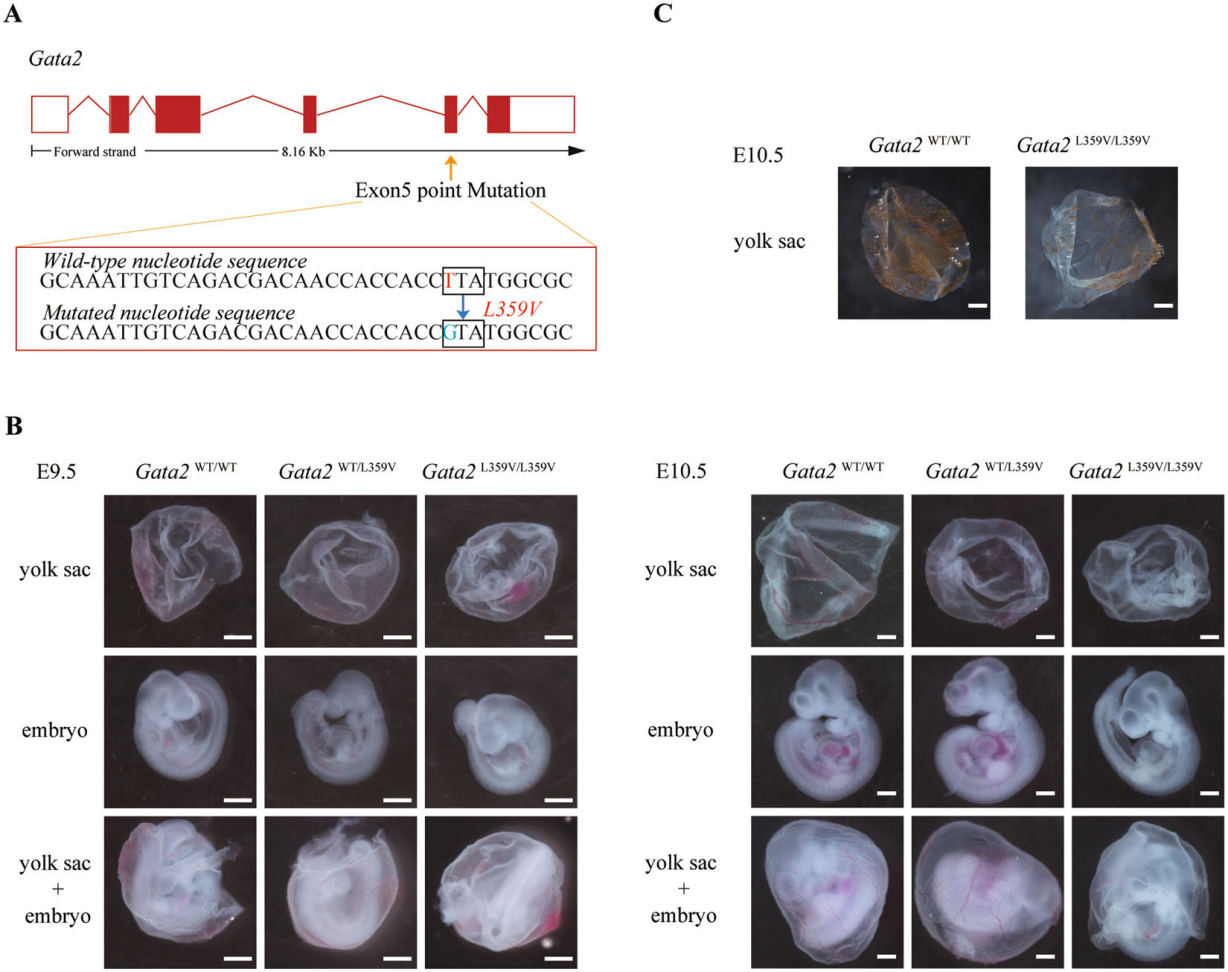
Anemia phenotype in *Gata2*^L359V/L359V^ embryos. **A** Schematic of the *Gata2-*L359V knockin murine model. The L359V mutation was introduced into the ZF2, which is located in the exon 5 of the murine *Gata2* gene. **B** Morphology of embryos and yolk sacs of different genotypes at E9.5 (left panel, scale bar 500 μm) and E10.5 (right panel, scale bar 500 μm). **C** *O*-dianisidine staining of hemoglobin in yolk sac erythrocytes (scale bar 500 μm).

To investigate the pathophysiological roles of *Gata2-*L359V mutation in vivo, we generated a *Gata2-*L359V knockin murine model (Fig. 1a and S1A, B). No homozygous (*Gata2*^L359V/L359V^) mutant was detected among over 20 littermates of heterozygous (*Gata2*^WT/L359V^) intercrosses (Figure S1C and Table S1), suggesting that *Gata2*^L359V/L359V^ mutants were embryonically lethal. The genotype of the embryo was identified by genomic DNA PCR. The time of homozygous embryonic death was determined using embryos from timed matings (Fig. S1D). At embryonic day (E) 10.5, all the genotypes of surviving embryos showed the expected Mendelian frequency. Besides, we also observed the genetic knockin did not alter the expression levels of WT or mutant Gata2 protein in embryos (Fig. S1E). However, no *Gata2*^L359V/L359V^ embryo survived beyond E11.5, whereas *Gata2*^WT/WT^ and *Gata2*^WT/L359V^ embryos remained viable at all embryonic stages (Table S1). These observations indicated that homozygous *Gata2-*L359V mutation resulted in midgestational embryonic lethality.

Next, we analyzed the embryos and yolk sacs, no morphological difference was observed among *Gata2*^L359V/L359V^, *Gata2*^WT/L359V^, and *Gata2*^WT/WT^ mutants at E9.5. However, *Gata2*^L359V/L359V^ embryos, which presented with pale yolk sacs, showed growth retardation at E10.5 (Fig. 1b). The o-dianisidine staining showed considerably decreased hemoglobin levels in *Gata2*^L359V/L359V^ yolk sacs at E10.5 (Fig. 1c), reflecting a decrease in erythropoiesis. These results indicated that homozygous mutation of *Gata2-*L359V induced severe anemia during mouse embryonic development.

### Homozygous *Gata2-*L359V mutation impairs embryonic erythroid differentiation

**Fig. 2:**
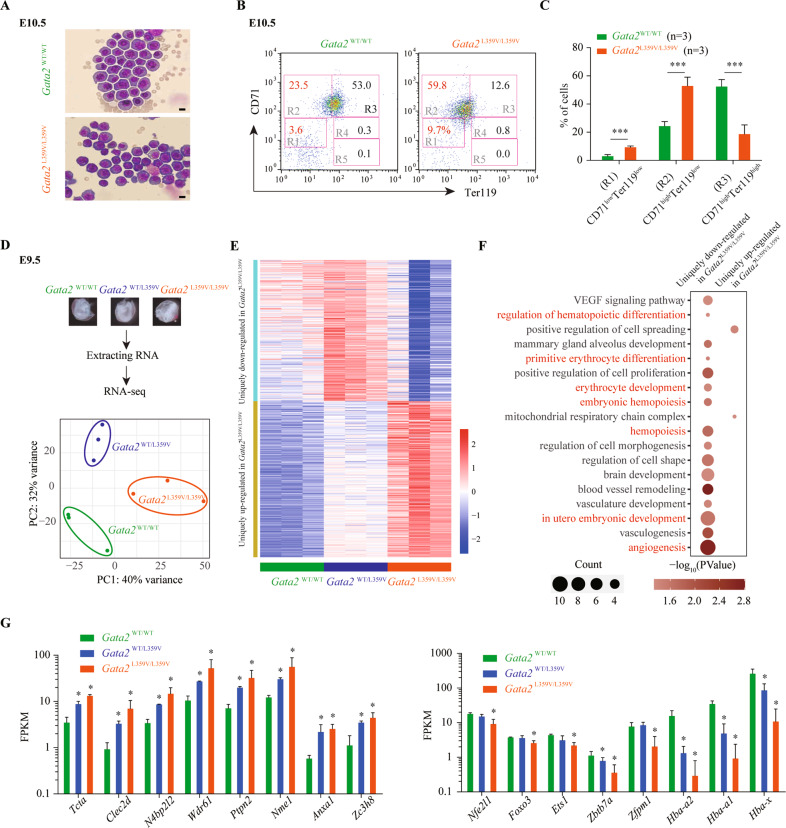
Erythropoiesis defects in *Gata2*^L359V/L359V^ embryos. **A** Morphological analysis of *Gata2*^WT/WT^ (upper panel) and *Gata2*^L359V/L359V^ (lower panel) yolk sac erythrocytes by using Wright’s staining (scale bar 10 μm). **B** Immunophenotype of E10.5 yolk sac cells marked by CD71/Ter119 and the cells were divided into five stages (R1-R5) following the expression levels of CD71 and Ter119. **C** Statistical analysis of the percentage of CD71^low^Ter119^low^, CD71^high^Ter119^low^, and CD71^high^Ter119^high^ cells between *Gata2*^WT/WT^ (*n* = 3) and *Gata2*^L359V/L359V^ (*n* = 3). **D** Principal component analysis (PCA) of the RNA-seq data of the *Gata2*^WT/WT^, *Gata2*^WT/L359V^, and *Gata2*^L359V/L359V^ yolk sacs at E9.5. **E** Heatmap showing a group of genes uniquely down-regulated or upregulated in *Gata2*^L359V/L359V^ as compared to *Gata2*^WT/L359V^ and *Gata2*^WT/WT^ E9.5 yolk sacs. **F** Gene ontology(GO) analysis of embryonic development genes that were uniquely up/down-regulated in *Gata2*^L359V/L359V^ as compared to *Gata2*^WT/L359V^ and *Gata2*^WT/WT^ E9.5 yolk sacs. **G** The fragments per kilobase million (FPKM) value of representative genes upregulated or down-regulated in *Gata2*^L359V/L359V^ E9.5 yolk sacs (*n* = 3, respectively). Error bars represent the standard deviation from the average. Significant differences are indicated by *p < 0.01 (Student’s *t* test).

To investigate the mechanism underlying anemia in *Gata2*^L359V/L359V^ yolk sacs, we determined the morphological features and immunophenotypes of *Gata2*^L359V/L359V^ and *Gata2*^WT/WT^ yolk sacs. Approximately 70% of the erythrocytes in E10.5 *Gata2*^L359V/L359V^ yolk sacs exhibited increased nucleus-to-cytoplasm ratio and dark blue-stained cytoplasm (Fig. 2a), suggesting impaired development of primitive erythropoiesis. We then performed immunophenotyping analysis by using antibodies against CD71 and Ter119. The embryonic erythroid development can be divided into five stages (designated as R1-R5), corresponding to different stages during erythroid differentiation^24^ (Fig. 2b). *Gata2*^L359V/L359V^ mutants showed significantly increased CD71^low^Ter119^low^ and CD71^high^Ter119^low^ precursors (R1 and R2 compartments) and decreased CD71^high^Ter119^high^ cells (R3 compartment; Figs. 2c and S2), indicating that homozygous *Gata2-*L359V mutation blocked embryonic erythropoiesis. Compared with erythroid marker CD71, the expression of myeloid markers and transcription factors were extremely low (Fig. S3A).

To further determine the program dysregulated by *Gata2-*L359V mutation during embryonic erythropoiesis, we performed RNA sequencing (RNA-seq) analysis in *Gata2*^L359V/L359V^, *Gata2*^WT/L359V^, and *Gata2*^WT/WT^ yolk sacs at E9.5 (Fig. 2d). A group of genes that were uniquely up/down-regulated in *Gata2*^L359V/L359V^ as compared to *Gata2*^WT/L359V^ and *Gata2*^WT/WT^ E9.5 yolk sacs were identified (Fig. 2e). With Gene ontology (GO) analysis, these genes were largely of functional relevance to embryonic hematopoiesis (e.g., erythrocyte development, hematopoietic progenitor cell differentiation, and primitive erythrocyte differentiation) and regulation of vascular development (e.g., vasculature development, angiogenesis, VEGF signaling pathway, blood vessel remodeling; Fig. 2f and Table S2). As shown in Fig. 2g, the expression levels of genes annotated for hematopoietic differentiation^25,26^, such as *Nfe2l1*^27^, *Ets1*^28^, *Zbtb7a*^29^, and *Zfpm1*^30^, were inhibited in *Gata2*^L359V/L359V^ group. Meanwhile, *Hba-a2*, *Hba-a1*, and *Hba-x* were down-regulated in *Gata2*^L359V/L359V^ sample, which was consistent with the anemia phenotype during the embryonic development (Figs. 2g and S3B). Additionally, we found some genes up/down-regulated in both *Gata2*^L359V/L359V^ and *Gata2*^WT/L359V^ as compared to *Gata2*^WT/WT^ group. GO analysis showed that genes related to certain metabolic pathways were upregulated, while those related to some important signal transduction pathways (such as Notch and Wnt pathways) were down-regulated in the two former groups (Fig. S3C), implying that although *Gata2*^WT/L359V^ mutants escaped from embryonic lethality, they might still carry subtle defects of physiological function due to the abnormal gene expression.

### Heterozygous *Gata2-*L359V mutation significantly affects hematopoietic reconstitution

*Gata2*^L359V/L359V^ mutants died around E11.5, but *Gata2*^WT/L359V^ mice remained viable. The peripheral blood (PB) cellular components, bone marrow (BM) cell morphological features, immunophenotypes, and frequencies of hematopoietic stem/progenitor cell (HSPC) subsets appeared to be normal (Fig. S4A–E), and the colony-forming capacities between *Gata2*^WT/WT^ and *Gata2*^WT/L359V^ BM cells showed no significant differences (Fig. S4F).

**Fig. 3:**
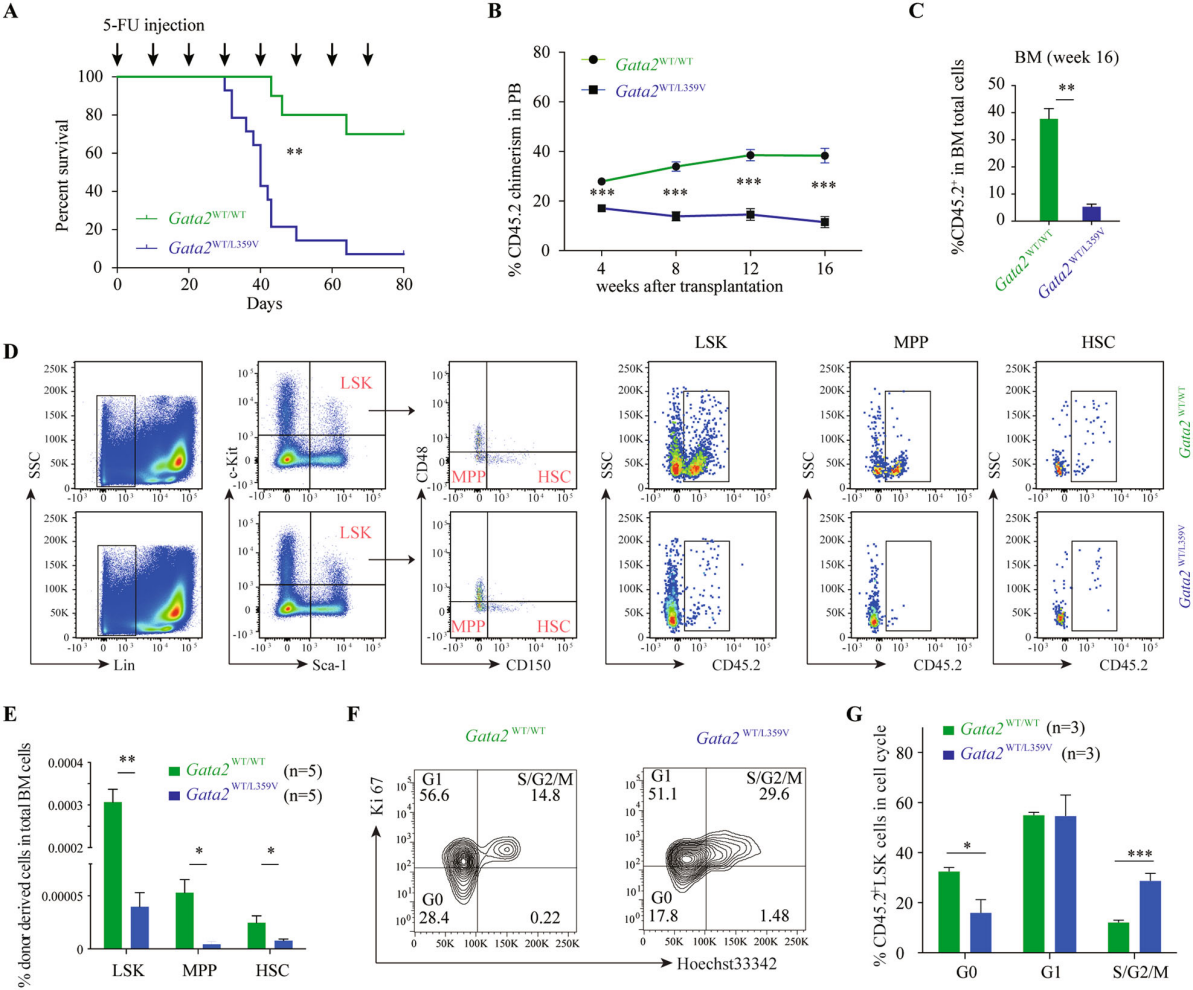
Impairment of definitive hematopoiesis in *Gata2*^WT/L359V^ mice. **A** Survival analysis of *Gata2*^WT/WT^ (*n* = 15) and *Gata2*^WT/L359V^ mice with 5-FU intraperitoneal injection. The 6–8-weeks-old *Gata2*^WT/WT^ (*n* = 15) and *Gata2*^WT/L359V^ (*n* = 15) mice were treated with 150 mg/kg 5-FU every 10 days until all the mice in one group die (*p* < 0.01, Kaplan–Meier analysis). **B** HSC reconstitution capacity analysis by competitive bone marrow (BM) transplantation. The reconstitution in recipient mice was followed for 16 weeks, and the percentages of CD45.2^+^ peripheral blood mononuclear cells (PBMCs) derived from *Gata2*^WT/WT^ (*n* = 19) and *Gata2*^WT/L359V^ (*n* = 17) donors were analyzed every 4 weeks (Student’s *t* test). **C** Statistical analysis of the percentage of CD45.2^+^ cells in the total BM cells between *Gata2*^WT/L359V^ (*n* = 5) and *Gata2*^WT/WT^ (*n* = 5) groups (Student’s *t* test) at 16 weeks after reconstitution. **D** *Gata2*^WT/L359V^ interfered with HSC self-renewal in competitive BM transplantation. Representative display of flow cytometry analysis was shown. The LSKs (Lin^-^c-Kit^+^Sca1^+^), MPPs (Multipotent progenitors, CD48^−^CD150^−^ LSK), and hematopoietic stem cell (HSCs, CD48^-^CD150^+^ LSK) were gated. The percentage of donor-derived cells (CD45.2^+^) were then examined. **E** Comparison of the percentage of donor-derived LSK, MPP, and HSC in reconstituted BM cells between *Gata2*^WT/L359V^ (*n* = 5) and *Gata2*^WT/WT^ (*n* = 5) groups (Student’s *t* test). **F** Representative display of cell cycle of LSKs from competitive BM transplants. BM cells were harvested at week-16 post-reconstitution and stained with Ki67 and Hochest33342. **G** Comparison of the cell cycle between *Gata2*^WT/L359V^ (*n* = 5) and *Gata2*^WT/WT^ (*n* = 5) groups (Student’s *t* test). Error bars represent the deviation from the average, and significant differences are indicated by *p < 0.05, **p < 0.01, and ***p < 0.001.

To have an in-depth investigation on the role of *Gata2-*L359V mutation in adult hematopoiesis, we treated *Gata2*^WT/L359V^ and *Gata2*^WT/WT^ mice with the cytotoxic drug 5-fluorouracil (5-FU) to examine the effect of this mutation on the hematopoietic recovery under stress condition^31,32^. The result showed that over 92.8% of *Gata2*^WT/L359V^ mice died, whereas only 30% of *Gata2*^WT/WT^ mice succumbed (Fig. 3a). We then performed competitive BM transplantation experiments to clarify whether *Gata2-*L359V mutation could interfere with HSC self-renewal. BM cells of the donor (CD45.2 *Gata2*^WT/WT^ and *Gata2*^WT/L359V^) and competitor mice (CD45.1) were mixed in the ratio of 1:1 and transplanted into recipient mice. Over the 16-week follow-up, the *Gata2*^WT/WT^ donor-derived cells increased from 27.89% to 38.32% in PB, whereas *Gata2*^WT/L359V^ donor-derived cells significantly decreased at all time points (from 17.07% to 11.50%; *P* < 0.01, Fig. 3b). An obvious decrease of *Gata2*^WT/L359V^ donor-derived cells was also observed in BM (Fig. 3c). Moreover, immunophenotype analysis revealed a remarkable reduction in the frequency of *Gata2*^WT/L359V^ donor-derived CD45.2^+^CD150^+^CD48^−^Lin^−^Sca-1^+^c-Kit^+^ (hematopoietic stem cells [HSCs]^33^) and CD45.2^+^CD150^−^CD48^−^Lin^−^Sca-1^+^c-Kit^+^ cells (multipotent progenitors [MPPs], this marker combination being chosen to avoid discrepancy in the literation on the definition of MPP^33–35^) in recipients, indicating that *Gata2-*L359V mutated HSCs were impaired in hematopoietic reconstitution under stress condition (Fig. 3d, e). Apart from the lesion of HSCs and MPPs, the numbers of *Gata2*^WT/L359V^ donor-derived downstream myeloid progenitors including common myeloid progenitor (CMP, Lin^−^Sca1^−^c-Kit^+^CD34^+^CD16/32^high^), granulocyte/monocyte progenitor (GMP, Lin^−^Sca1^−^c-Kit^+^CD34^+^CD16/32^low^), and megakaryocyte/erythroid progenitor (MEP, Lin^−^Sca1^−^c-Kit^+^CD34^−^CD16/32^−^) were also decreased (Fig. S5G). To better understand these reconstitution defects, we carried out cell cycle analysis on BM Lin^−^Sca-1^+^c-Kit^+^ (LSK) cells at week-16 post-transplantation. The percentage of *Gata2*^WT/L359V^ donor-derived LSKs at G0 phase was dramatically decreased, whereas the percentage of that at S/G2/M phase was significantly increased compared with the control group (Fig. 3f, g), indicating the effect of *Gata2-*L359V on HSC exhaustion.

### Heterozygous *Gata2*-L359V mutation induces subtle molecular alteration in definitive hematopoiesis

**Fig. 4:**
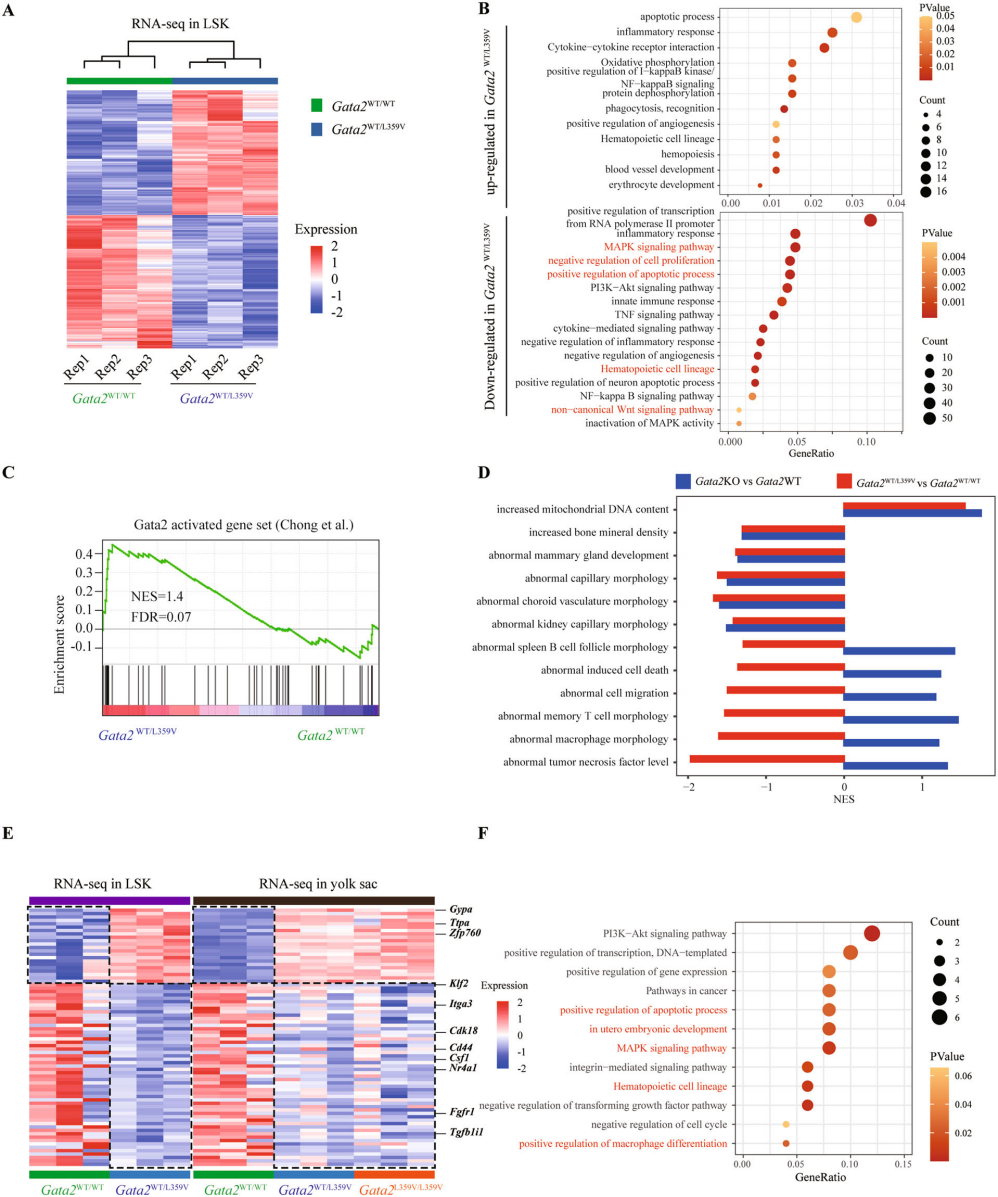
Molecular defect of *Gata2-*L359V in definitive hematopoiesis. **A** Heatmap showing the differentially expressed genes in the LSKs of *Gata2*^WT/L359V^ as compared with *Gata2*^WT/WT^ group. Every sample was comprised of an LSK mixture from three mice of the same genotype. **B** GO analysis of genes up/down-regulated in *Gata2*^WT/L359V^ compared to *Gata2*^WT/WT^ LSKs. **C** Gene set enrichment analysis (GSEA) of Gata2 activated gene set in *Gata2*^WT/L359V^ vs *Gata2*^WT/WT^ LSKs. **D** Enrichment analysis of gene sets of mammalian phenotype ontology in *Gata2*KO vs *Gata2*WT or *Gata2*^WT/L359V^ vs *Gata2*^WT/WT^. **E** Heatmap showing genes differentially expressed between *Gata2*^WT/L359V^ and *Gata2*^WT/WT^ in both yolk sacs and LSKs. **F** GO analysis of genes down-regulated in *Gata2*^WT/L359V^ vs *Gata2*^WT/WT^ in both yolk sacs and LSKs.

To explore the molecular mechanism underlying HSC defects, we performed the RNA-seq on BM LSKs from 8-week-old *Gata2*^WT/WT^ and *Gata2*^WT/L359V^ mice under steady-state conditions (Table S3). Unsupervised hierarchical clustering revealed the difference in gene expression profiles between two groups (Fig. 4a). GO analysis showed that some genes related to negative regulation of cell cycle and proliferation (such as *Klf4*, *Osm*, and *Nr2e3*), positive regulation of apoptotic process (such as *Tnf*, *Gadd45b*, and *Bcl211*), Wnt signaling pathways (such as *Wnt11*, *Fzd4*, and *Fzd8*), and MAPK signaling pathway (such as *Fgf3*, *Fos*, and *Hspa1b*) were down-regulated in the LSKs of *Gata2*^WT/L359V^ as compared with those in *Gata2*^WT/WT^ group (Fig. 4b and Table S3). However, when some genes essential for HSC self-renewal such as *Spi1*^36^, *Tet2*^37^, *Dnmt3a*^38^, and *Mllt3a*^39^ were examined, no significant difference were observed (Fig. S5A), which might be ascribed to the inability of heterozygous Gata2 to induce dramatically transcriptomic changes under steady status. This observation also supported the lack of significant difference of the HSC and lineage bias between *Gata2*^WT/L359V^ and *Gata2*^WT/WT^.

Furthermore, we combined our transcriptome dataset of *Gata2*^WT/L359V^ with that of heterozygous *Gata2*KO LSKs reported previously^21,22,40^. Genes activated by Gata2 tended to be enriched in *Gata2*^WT/L359V^ versus *Gata2*^WT/WT^ LSKs (FDR = 0.07)^21,22^ (Fig. 4c). Gene set enrichment analysis (GSEA) showed that although heterozygous *Gata2*-L359V and *Gata2*KO had opposite effects on some pathways involved in cell function, they showed similar expression patterns in many other important pathways (Fig. 4d). Besides, it has been reported that *Gata2* overexpression inhibited genes involved in angiogenesis and endothelial cell differentiation^41^. Consistent with this report, our GSEA analysis also showed that gene sets of angiogenesis and endothelial cell differentiation were significantly down-regulated in *Gata2*^WT/L359V^ LSKs (Fig. S5B). Taken together, our data suggested that *Gata2*-L359V mutation exerts complex transcriptional regulatory function. In addition, we compared the RNA-seq data between BM LSKs and yolk sacs. Indeed, *Gata2*-L359V down-regulated some genes related to hematopoietic cell lineage^25,26^ (such as *Csf1*, *Itga3*, and *Cd44*) and in utero embryonic development^25,26^ (such as *Meg3*, *Syvn1*, *Klf2*, and *Fgfr1*) in both BM LSKs and yolk sacs (Fig. 4e, f).

### Gata2*-*L359V acquires enhanced chromatin-binding ability and transcriptional activity

**Fig. 5:**
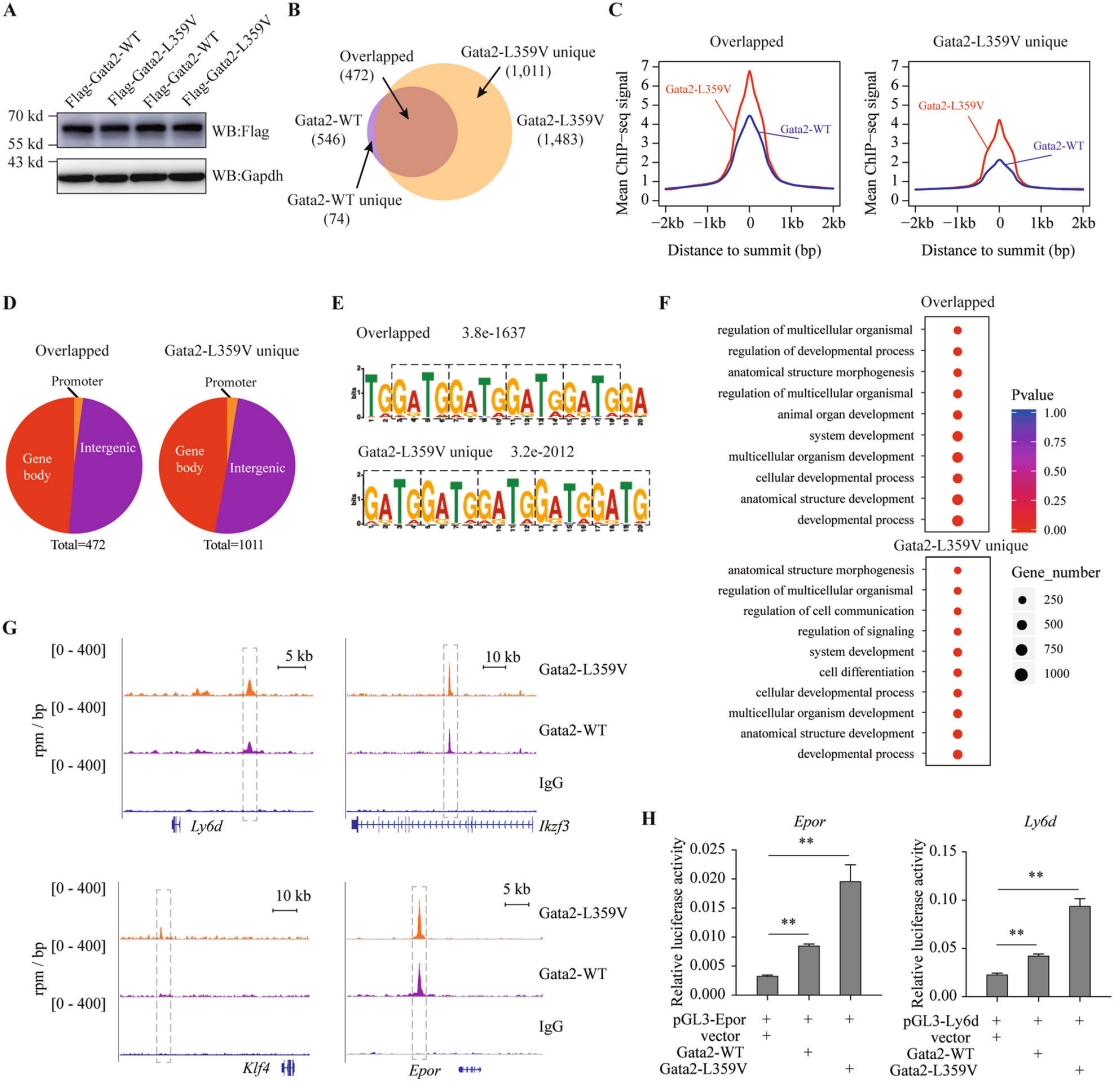
Enhancement of DNA binding and transcriptional regulation on Gata2 targets by *Gata2*-L359V mutation. **A** Validation of the enforced expression of Gata2-WT or Gata2-L359V in 32D cells using an antibody that can recognize different species of Gata2 protein, including WT and Gata2-L359V mutant forms. The Flag-Gata2-WT or Flag-Gata2-L359V plasmid were transfected into the 32D cells. Antibodies against Flag or Gaphd were used for western blot analysis. **B** Overlap analysis between Gata2-WT- and Gata2-L359V-bound regions. The Gata2-WT uniquely bound (Gata2-WT unique), Gata2-L359V uniquely bound (Gata2-L359V unique), and Gata2-WT/Gata2-L359V co-bound (Overlapped) targets were identified. **C** ChIP-seq binding signal of Gata2-WT and Gata2-L359V on the overlapped and the Gata2-L359V unique regions. **D** Genomic distribution patterns of the overlapped and the Gata2-L359V unique regions. **E** De novo motif analysis of the overlapped and the Gata2-L359V unique regions. **F** GO analysis of genes in the overlapped regions or Gata2-L359V unique bound regions. **G** Genome browser visualization of Gata2-WT and Gata2-L359V binding on the representative targets. **H** Analysis of Gata2-WT and Gata2-L359V on the enhancer activity of *Ly6d* and *Epor* (*p* < 0.01, Student’s *t* test).

To explore the mechanism underlying the *Gata2-*L359V mutation-mediated gene dysregulation, we ectopically expressed Flag-Gata2-L359V and Flag-Gata2-WT in 32D cells^42^ (Fig. 5a), a murine myeloid precursor cell line, and performed chromatin immunoprecipitation sequencing (ChIP-seq). By this approach, we preliminarily identified 546 Gata2-WT- and 1483 Gata2-L359V-bound regions (Table S4), and most of Gata2-WT bound regions were also bound by Gata2-L359V (Fig. 5b). We then compared the binding signals of Gata2-L359V and Gata2-WT and identified 74 Gata2-WT uniquely bound regions, 1011 Gata2-L359V uniquely bound regions, and 472 Gata2-WT and Gata2-L359V co-bound regions. Intriguingly, on the overlapping regions, the binding signals of Gata2-L359V were significantly higher than those of Gata2-WT (Fig. 5c). On the Gata2-L359V unique regions, nevertheless, Gata2-WT still had weak binding signals. This result implied that *Gata2*-L359V mutation might not alter the Gata2 targets but could significantly enhance the binding affinity on these targets.

We next compared the genomic distribution patterns of these regions and found that the overlapping and Gata2-L359V unique regions were distributed in a similar way (Fig. 5d). Both WT and mutated Gata2 scattered widely across the whole genome, including promoter, gene body, and intergenic regions, as previously reported^43^. The motif analysis on the overlapping regions and the Gata2-L359V unique regions showed that Gata2-L359V and Gata2-WT both bound to the GATA/G repeat sequences (Fig. 5e). As previously reported, GATG motifs can also serve as binding sites for GATA2 besides the canonical GATA motifs^44^. Subsequently, we performed GO analysis of the genes in overlapping and Gata2-L359V unique regions. It was found that a number of Gata2-L359V unique genes were related to cell differentiation (Fig. 5f), which might partially account for the abnormal hematopoietic differentiation in our model systems. The binding signals of Gata2-L359V and Gata2-WT on four representative targets were illustrated in Fig. 5g, including *Epor* (one of the master regulators of erythrocyte differentiation^41,45^), *Klf4*, *Ikzf3* (both known to be crucial for cell differentiation and stemness regulation^46,47^), and *Ly6d* (a specification marker of lineage commitment^48^). In line with the genome browser visualization, luciferase reporter assay showed that, though both Gata2-L359V and Gata2-WT could activate the transcription of *Epor* and *Ly6d*, the activation by Gata2-L359V was much stronger (Fig. 5h).

We also performed an integrative analysis of the ChIP-seq and RNA-seq data. Amongst Gata2-L359V unique bound genes, *Bcas3*, *Sall4*, and *Vegfa*, annotated to embryonic lethality and abnormal hematopoiesis according to mammalian phenotype ontology^26^, showed different expression patterns between *Gata2*^L359V/L359V^ and *Gata2*^WT/L359V^ yolk sacs (Fig. S6A). As for Gata2-L359V unique bound genes in BM LSKs, hematopoietic transcription factor *Klf4*, *Klf6*, and *Jun* showed a significantly lower expression, while the expression of cell cycle-related gene *Ccnd1* was much increased, in *Gata2*^WT/L359V^group than in *Gata2*^WT/WT^ one (Fig. S6B). These results implied that Gata2-L359V mutant might acquire the capacity to suppress the stemness of HSCs and promote cell cycle potentially through dysregulating the transcription of the key factors.

Meanwhile, we analyzed the overlap of the targeted genes between Gata2-L359V unique and previously reported GATA1/GATA2 datasets^49^. Amongst 2524 genes bound by both GATA1 and GATA2, 133 genes were overlapped with Gata2-L359V unique targets. GO analysis revealed that these genes were functionally involved in biological processes including cell differentiation, development, signal transduction, and metabolism (Fig. S6C). It implied that Gata2-L359V mutant might interfere with the GATA switch likely through these unique binding regions across the three settings.

### *Gata2-*L359V mutation disturbs the differentiation of *BCR/ABL-*induced murine CML cells

**Fig. 6:**
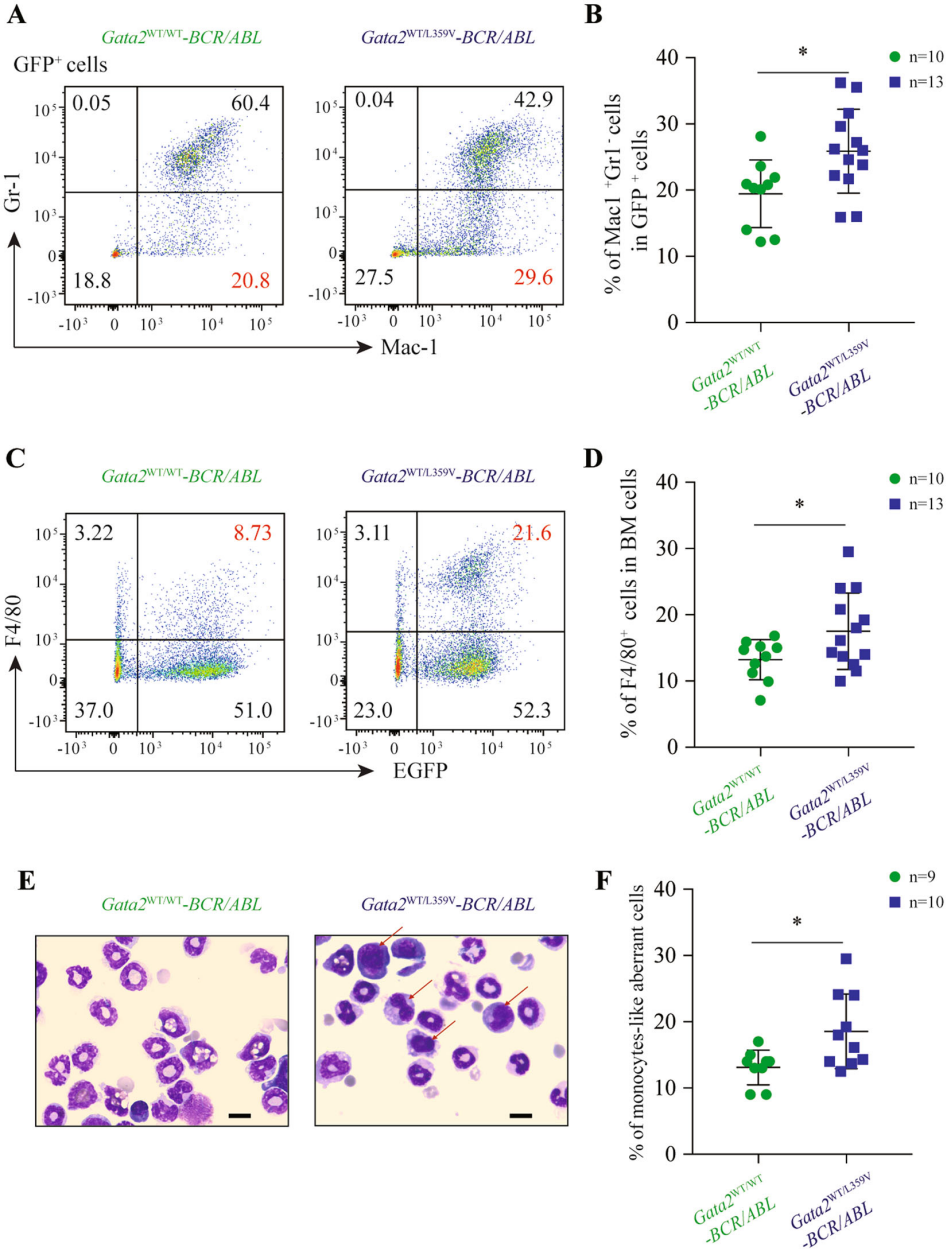
Cell differentiation interference by *Gata2*-L359V mutation in a murine model of chronic myeloid leukemia. **A** Representative display of flow cytometry analysis of Gr-1^−^Mac-1^+^ cells in GFP^+^ BM cells. **B** Statistical analysis of Gr-1^−^Mac-1^+^ cell frequencies between *Gata2*^WT/WT^-*BCR/ABL* (*n* = 10) and *Gata2*^WT/L359V^-*BCR/ABL* (*n* = 13) mice. **C** Representative flow cytometry analysis of GFP^+^F4/80^+^ cells in total BM cells. **D** Statistical analysis of GFP^+^F4/80^+^ cell frequencies between *Gata2*^WT/WT^-*BCR/ABL* (*n* = 10) and *Gata2*^WT/L359V^-*BCR/ABL* (*n* = 13) mice. **E** Morphological analysis of BM cells in *Gata2*^WT/WT^-*BCR/ABL* (*n* = 10) and *Gata2*^WT/L359V^-*BCR/ABL* (*n* = 13) mice. BM cells were harvested and subject to Wright’s staining. The red arrows represent the atypical monocytes (scale bar 10 μm). **F** Statistical analysis of the percentage of monocyte-like aberrant cells in BM between *Gata2*^WT/WT^–*BCR/ABL* (*n* = 9) and *Gata2*^WT/L359V^-*BCR/ABL* (*n* = 10) mice. Error bars represent the deviation from the average, and significant differences are indicated by *p < 0.05 (Student’s *t-*test).

Considering that *Gata2-*L359V mutation was identified in the CML blast crisis^22,50^, we used a murine CML model to determine the role of this mutation in differentiation blockage. *Gata2*^WT/WT^ and *Gata2*^WT/L359V^ BM cells were infected with the *BCR/ABL*-expressing retrovirus and then injected into the lethally irradiated recipients^51,52^. The BM immunophenotype analysis revealed a CML-like disease characterized by the massive expansion of mature granulocytes (GFP^+^/Gr-1^+^/Mac-1^+^ cells) in both groups (Fig. 6a). Notably, the number of Gr-1^−^/Mac-1^+^ monocytic cells in the *Gata2*^WT/L359V^-*BCR/ABL* group (25.88% ± 1.75%) was significantly higher than that in the *Gata2*^WT/WT^-*BCR/ABL* group (19.44% ± 1.61%; *p* = 0.0158; Fig. 6b). The F4/80^+^ monocytic cells in *Gata2*^WT/L359V^-*BCR/ABL* mice (17.51% ± 1.60%) were also higher than those in *Gata2*^WT/WT^-*BCR/ABL* mice (13.21% ± 0.95%; *p* = 0.0448; Fig. 6c, d). The BM cells of representative mice were collected and subjected to Wright’s staining, showing the granulocytes significantly increased in both groups (Fig. 6e). However, in addition to the basic CML-like phenotype, a group of monocyte-like aberrant cells with irregularly shaped nuclei and dark blue cytoplasm was noticeable in the *Gata2*^WT/L359V^-*BCR/ABL* group (Fig. 6f). Similar to the previous findings^51–53^, *Gata2*^WT/L359V^-*BCR/ABL* mice showed a longer life span than *Gata2*^WT/WT^-*BCR/ABL* mice (Fig. S7).

### Molecular signature alteration reveals the *Gata2-*L359V mutation as a driver for the increment of GMPs and monocytosis in *BCR/ABL*-induced CML model

**Fig. 7:**
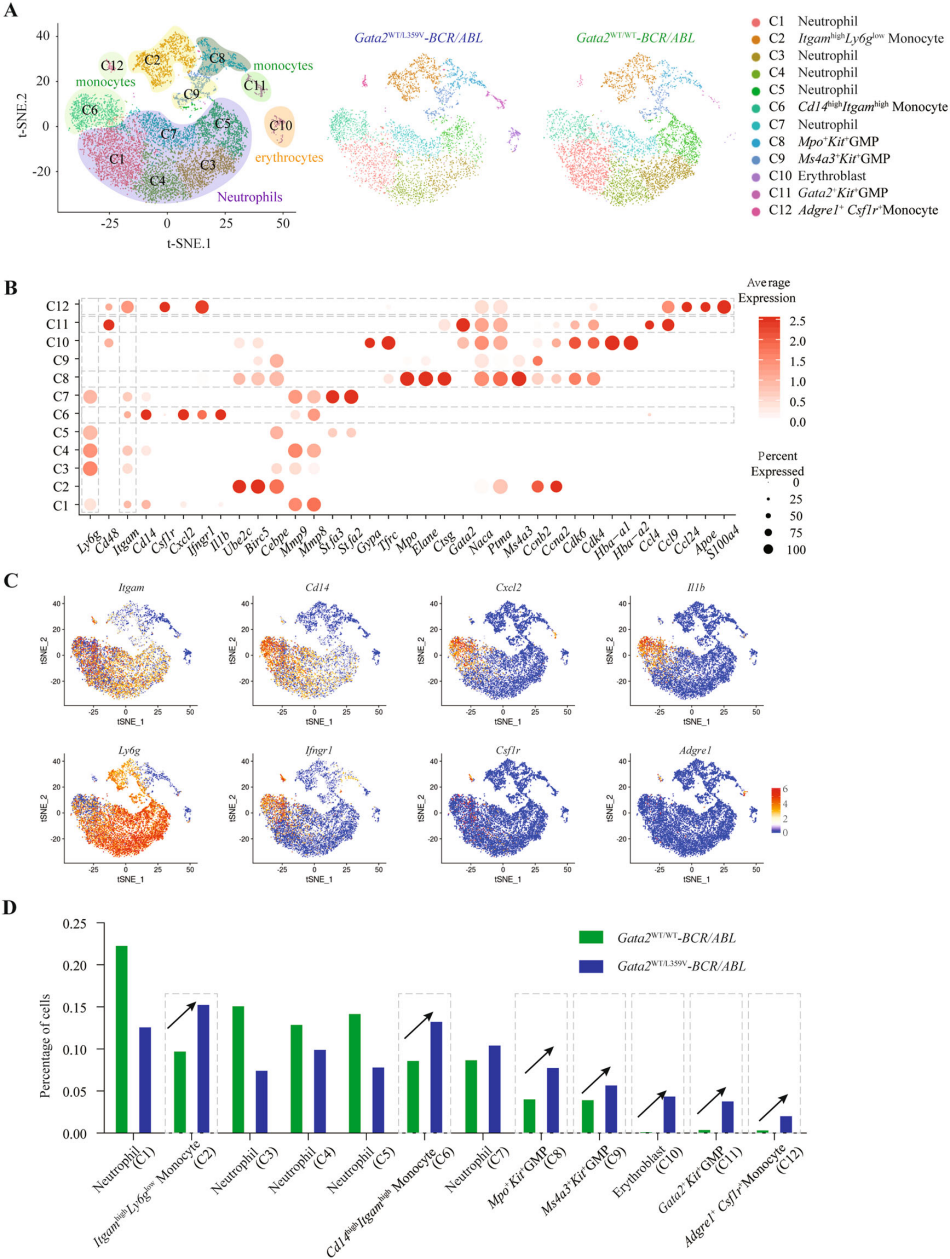
Dynamic changes between leukemic cells of *Gata2*^WT/L359V^-*BCR/ABL* and *Gata2*^WT/WT^-*BCR/ABL* mice revealed by single-cell RNA-seq analysis. **A** t-distributed Stochastic Neighbor Embedding (t-SNE) showing 12 clusters of BM cells from *Gata2*^WT/L359V^-*BCR/ABL* and *Gata2*^WT/WT^-*BCR/ABL* mice. The left panel showed the 12 clusters of the merged scRNA-seq datasets. The middle panels showed the separated illustration of the t-SNE map in *Gata2*^WT/L359V^-*BCR/ABL* and *Gata2*^WT/WT^-*BCR/ABL* group. The right panel showed the classification of the cell types. **B** Expression analysis of the cluster-specific genes. Dot plot showing the expression of representative genes across the 12 classes of cells. **C** t-SNE showing the expression of representative genes in different classes of cells. **D** Comparison of the percentage of each class of cells between *Gata2*^WT/L359V^-*BCR/ABL* and *Gata2*^WT/WT^-*BCR/ABL* BM.

To compare the transcriptomic characteristics of BM cells between *Gata2*^WT/L359V^-*BCR/ABL* and *Gata2*^WT/WT^-*BCR/ABL* mice, we performed single-cell RNA-seq (scRNA-seq) in two representative mice. A total of 7787 single cells were analyzed from which we identified 12 classes of cell types^54,55^ (Figs. 7a and S8A). Consistent with the murine CML phenotype, most of the BM cells in *Gata2*^WT/L359V^-*BCR/ABL* and *Gata2*^WT/WT^-*BCR/ABL* mice were neutrophils (C1, C3–C5, and C7). Monocytes, macrophages, erythroid progenitors, and GMPs were also noted. Three classes (C2, C6, and C12) of monocytes were characterized by *Itgam*^high^*Ly6g*^low^, *Cd14*^high^*Itgam*^high^, and F4/80(*Adgre1*)^+^*Csf1r*^+^, respectively, and three classes (C8, C9, and C11) of GMPs were characterized by *Gata2*^+^*Kit*^+^, *Ms4a3*^+^*Kit*^+^, and *Mpo*^+^*Kit*^+^, respectively, which represented distinct stages along with the monocytic differentiation^56,57^ (Fig. 7b, c). We then compared the ratio of each cell type in BM from *Gata2*^WT/L359V^-*BCR/ABL* or *Gata2*^WT/WT^-*BCR/ABL* mice and found that the three classes of monocytes and a small subgroup of erythrocytic progenitors (C10) were increased in the former group. Notably, the percentages of C11, C9, and C8 GMPs were 0.4%, 3.9%, and 4.0%, respectively in the *Gata2*^WT/WT^-*BCR/ABL* vs 3.8%, 5.6%, and 7.7%, respectively in the *Gata2*^WT/L359V^-*BCR/ABL* group, indicating an increment of progenitors in the *Gata2-*L359V setting. Besides, the percentages of C2, C6, and C12 monocytes were 9.7%, 8.5%, and 0.3%, respectively in *Gata2*^WT/WT^-*BCR/ABL* vs 15.2%, 13.2%, and 2.0%, respectively in *Gata2*^WT/L359V^-*BCR/ABL* group (Fig. 7d). Our observation of the increase in the BM percentages of GMPs and monocytes suggested that the leukemic cell mass was, at least in part, directed to the monocytic precursors dysregulated by *Gata2-*L359V mutation on the basis of *BCR/ABL*-induced CML (30.3% vs 18.5%). When scRNA-seq data were subject to further analysis, pathways related to myeloid/leukocyte differentiation and function were found dysregulated in GMPs of *Gata2*^WT/L359V^-*BCR/ABL* mouse (Fig. S8B). Moreover, Pu.1 targets were observed down-regulated in the neutrophils and *Csf1r*^+^ monocytes of *Gata2*^WT/L359V^-*BCR/ABL* BM (Fig. S9), supporting the interference of Pu.1 function upon the effect of Gata2-L359V as previously reported by biochemical approach^25^. These data suggested that Gata2-L359V partially impeded the cell differentiation at the early stage of myelomonocytic lineage, thus promoting the CML progression.

## Discussion

GATA2 is a master zinc-finger transcription factor and plays essential roles in the regulation of HSC activity and stimulation of myeloid-erythroid progenitor differentiation under physiological conditions^58,59^. In the present work, we have provided further evidence for the pivotal function of Gata2 in the regulation of primitive and definitive hematopoiesis with a murine *Gata2*-L359V knockin model. RNA-seq data and integrative analysis of available gene expression datasets in relevant cell/tissue systems allowed us to address the molecular networks underlying the major phenotypic features at distinct development stages. Meanwhile, by using scRNA-seq, we showed that heterozygous *Gata2-*L359V mutation induced an increased number of GMPs associated with monocytic lineage expansion in *BCR/ABL*-transduced CML model.

It has been well established that primitive hematopoiesis is key to the murine embryonic development at E7.5^60,61^. Embryos with defects of the primitive hematopoiesis cannot develop beyond E11.5. Then the definitive hematopoiesis, originated from aorta-gonad-mesonephros (AGM) region of the embryo, takes place in fetal liver and BM to ensure the functions of blood system through fetal life to after birth. The downregulation of *Gata2* induced by gene KO or the deletion of a cis-regulatory element of *Gata2* resulted in embryonic lethality with severe anemia at E10.5^10,58^. Intriguingly, the *Gata2*^L359V/L359V^ embryos showed a similar death outcome and deficient erythroid differentiation around E11.5. In concordance, genes involved in embryonic hematopoiesis and other development processes were dysregulated in *Gata2*^L359V/L359V^ but not in *Gata2*^WT/L359V^ yolk sac. Nevertheless, compared to *Gata2*^WT/WT^, the *Gata2*^WT/L359V^ embryos still harbored distinct expression profiles of genes related to some important signal transduction, heralding a potential non-lethal defect in the heterozygous mutants.

Indeed, during adult life, although *Gata2*^WT/L359V^ mice displayed no obvious abnormality under steady-state conditions, they showed dramatically reduced BM reconstitution capacity under stress, likely due to the exhaustion of the HSC quiescent pool. RNA-seq data showed that the expression of some transcription factors regulating hematopoietic differentiation was down-regulated whereas the expression levels of genes promoting cell cycle and proliferation were increased in *Gata2-*L359V mutants, which could impair HSC and MPP functions. Application of serial BM transplantation experiment with improved technology in the future may further define the impact of *Gata2*-L359V on long-term HSCs. Growing evidence has demonstrated that mice lacking Gata2 exhibited a defect in definitive hematopoiesis^6,9^, and the haploinsufficiency of *GATA2* perturbs HSC homeostasis^6,9,59^. The overexpression of GATA2 also suppresses hematopoiesis^11,62^. Hence, the integrity of the Gata2 associated regulatory network is indispensable for a balanced self-renewal/differentiation potential of HSCs during adult hematopoiesis.

An interesting finding of the present work is the enhanced DNA-binding capacity of Gata2-L359V than Gata2-WT in ChIP-seq analysis. The core DNA binding preference of GATA2 is the ‘GAT’ motif, whereas the well-characterized DNA-binding site of GATA2 is the ‘GATAA’ motif identified in the regulation of erythropoietic differentiation^63,64^. In fact, the motifs of GATA2 may vary in distinct cell types. Different from the canonical GATAA motif, Gata2-WT and Gata2-L359V in 32D cells were found to preferentially bind to the ‘GATG’ repeat sequence, implying that the mechanism underlying the regulation of Gata2 on early hematopoietic differentiation in the context of 32D cell line might be slightly different from that on terminal erythropoietic differentiation. Taken together, our data suggest that the Gata2 level and appropriate target occupancy must be constrained within a physiological window, while its insufficient or excessive activity and/or scope could impair hematopoiesis^65^. On the other hand, Gata2-L359V should be considered as an aberrant transcription factor with complex functions instead of a simple “gain-of-function” mutation. A study focusing on the GATA switch is also warranted in the future for understanding the mechanism behind the dysregulation of Gata2-L359V on hematopoiesis at a distinct dimension.

The *Gata2-*L359V mutation has been initially identified by our group as an aberrant transcription factor in cooperation with *BCR/ABL* fusion gene in CML patients with myelomonoblastic transformation^25^. By using murine BM transplantation model, *Gata2-*L359V, and *BCR/ABL* co-transduction led to an increase of BM monocytes^22^. In contrast to the human course of CML and other murine AML model, CML-like disease induced by retroviral *BCR/ABL* transfer in mice often deteriorates rapidly due to capillary embolism caused by excessive mature granulocytes in vital organs such as the lungs. Paradoxically, the burden of the peripheral embolism was relieved in CML blast-crisis mice by the blockage of cell differentiation, leading to a longer survival^51–53^. Consistent with this, *Gata2*^WT/L359V^*-BCR/ABL* mice also lived longer than the *Gata2*^WT/WT^*-BCR/ABL* controls. By performing scRNA-seq analysis, we identified 12 different t-distributed stochastic neighbor embedding (tSNE) clusters from leukemic cells based on their transcriptomic signature, which allowed the detection of subtle differences among distinct cell types at the molecular level. Flow cytometry or Cytometry by Time of Flight (CyTOF) analysis might be conducted to further validate the cell features in the future. Also, studies on the biological function of these subsets of cells might be conducted to explore the *Gata2-*L359V mediated abnormality in CML. Moreover, we found that *Gata2-*L359V suppressed the expression of Pu.1 targets in neutrophils and *Csf1r*^+^ monocytes by using the scRNA-seq data, supporting our previous in vitro study that *Gata2* L359 could interfere with Pu.1 function. These results have further enriched our understanding of the activity of *Gata2-*L359V in blocking the differentiation of *BCR/ABL*-expressing CML stem/progenitor cells, thus helping us comprehend the stepwise pathogenesis in this unique disease model.

## Materials and methods

### Mice experiments

All animal experiments were conducted following the institutional ethical guidelines on animal care and approved by the Department of Animal Experimentation of the Shanghai Jiao Tong University School of Medicine. The *Gata2-*L359V knockin murine model was constructed by the Model Animal Research Center of Nanjing University, China. The L359V mutation was introduced into the ZF2 domain located in the exon 5 of the murine *Gata2* gene with a PGK-neo cassette in the nearby intron (Figs. 1a and S1A). The *Gata2* target fragment was cloned into the pMD-18T vector, followed by point mutagenesis of L359V. Meanwhile, the *Gata2* target fragment on the BAC plasmid was replaced by the rpsL-neo cassette using gene recombination, and then the rpsL-neo cassette was replaced by the nonselectable fragment containing L359V mutation. The fragment containing the 5 kb 5′ arm, mutation point, and 5 kb 3′ arm was retrieved to the retrieving vector PL253 by gene recombination, and the PGK-neo cassette was subsequently inserted into the intron near the mutation point as a selective marker. The PL253 vector was then linearized at the 5′ end and electroporated into ES cells. G418-resistant ES cell clones were identified using Southern blot and injected into blastocysts to generate chimeric mice. Allele-specific primer sets were designed to distinguish between wild-type and mutated genotypes and were listed in Table S5. All experiments were performed in C57BL/6 mice except the CML model, which was built in mice on BALB/c background through backcross breeding.

### Embryo morphology

Yolk sacs were fixed in methylcellulose and observed under a stereomicroscope (Nikko ECLIPSE TS100). The survival of embryos was determined by heartbeat or embryo dissolution. The PECAM-1 staining was performed using monoclonal antibody MEC13.3 and detected using an HRP Detection Kit (BD Bioscience). Hemoglobin was stained with *o*-dianisidine (Sigma-Aldrich). The erythrocytes from the yolk sacs were stained using Wright’s stain (Sigma-Aldrich) and photographed under a microscope (Olympus BX61TRF).

### 5-FU treatment

*Gata2*^WT/WT^ and *Gata2*^WT/L359V^ mice aged 6–8 weeks received a single intraperitoneal injection of 150 mg/kg 5-FU (Sigma-Aldrich). The PB mononuclear cells were counted every three days. Additionally, *Gata2*^WT/WT^ and *Gata2*^WT/L359V^ mice were injected with 150 mg/kg 5-FU every 10 days for survival curve analysis.

### Competitive BM transplantation

A total of 40 male C57BL/6 mice aged 6–8 weeks were used in competitive transplantation. The BM cells isolated from CD45.2 mice (5 × 10^5^ cells) were mixed with an equivalent number of cells from CD45.1 mice and transplanted into lethally irradiated CD45.1 WT recipients. The percentage of CD45.2^+^ cells in PB of the engrafted recipients were tested every 4 weeks, and BM cells were harvested at 16 weeks after transplantation. The immunophenotype of BM cells was analyzed via flow cytometry by using the mouse lineage antibody cocktail and antibodies against c-Kit, Sca-1, CD150, CD48, CD16, CD32, CD34, and CD45.2. Cells were stained, fixed, and permeated following the manufacturer’s instruction of the Transcription Factor Buffer Set (BD Pharmingen™, 562574).

### Flow cytometry, western blot, and real-time qPCR experiments

Flow cytometry was performed on the FACSLSRII flow cytometers (BD Biosciences) and analyzed using the FlowJo Software (version 9.3.2). All antibodies were purchased from BD Biosciences. The western blot experiments were described as previously described^66,67^. Antibodies against the N-terminals of Gata2 were used for the detection of the embryonic Gata2 expression. Antibodies against Flag were used for the detection of Flag-tagged Gata2 proteins in 32D cells. Antibodies against Gapdh were used as the internal control. RT-qPCR was performed to validate the RNA-seq results. Total RNA was extracted as described above, and cDNA was synthesized using the PrimeScript® RT reagent Kit (TaKaRa Biotechnology Co. Ltd.). Real-time qPCR was performed using the primers listed in Table S5.

### Retroviral transduction and BM transplantation

Retrovirus generation and BM transplantation were performed as previously described^51,52^. Briefly, a total of 50 male BALB/c mice aged 6–8 weeks were used in retroviral transduction and BM transplantation. BM cells were isolated from the donor mice pretreated with 5-FU (250 mg/kg) and infected with retroviruses containing *MigR1-BCR/ABL* once daily for 2 days in transplant medium. In all, 5 × 10^5^ cells per mice were transplanted into the irradiated (3.4 Gy twice at a 3-h interval) recipient mice through tail vein injection randomly. After 3 weeks of transplantation, BM cells were subjected to morphological examination, flow cytometry analysis, and scRNA-seq analysis. For morphological examination, cells were centrifuged onto a glass slide and subjected to Wright’s staining (Sigma-Aldrich). Light microscopy images were obtained using the Nikko ECLIPSE TS100.

### RNA-seq and data analysis

The RNA-seq experiments were conducted as previously reported^67^. E9.5 yolk sacs dissected under a microscope were used for RNA-seq analysis. For each genotype (*Gata2*^WT/WT^, *Gata2*^WT/L359V^, and *Gata2*^L359V/L359V^), three yolk sacs were used as biological replicates. The DNA and RNA were simultaneously isolated with the All Prep DNA/RNA Mini Kit (Qiagen) according to the manufacture’s instruction. The DNA sample was sent for genotyping and RNA samples were used for RNA-seq analysis. LSK cells isolated from the BM of 6- to 8-week-old *Gata2*^WT/WT^ and *Gata2*^WT/L359V^ male mice were used for RNA isolation. Three biological replicates were carried out for each genotype (*Gata2*^WT/WT^ and *Gata2*^WT/L359V^), and three individual BM of the same genotype were mixed as one biological sample. RNA-sequencing libraries were constructed with the SMARTer® Universal Low Input RNA Kit for Sequencing according to the manufacture’s instruction. The libraries were sequenced with the Illumina MiSeq.

The RNA-seq data in FASTQ format was mapped against the mouse genome (mm10) using the STAR (v2.7.0)^68^, and the counts of each gene were calculated using HTseq (v0.6.1)^69^. The gene annotation files were downloaded from the UCSC Genome Browser (http://hgdownload.soe.ucsc.edu/). The significantly differentially expressed genes between different conditions were obtained using the DEseq2^70^ with a cutoff of the adjusted *p*-value (FDR) <0.05 and |Log_2_(Fold Change)| > 0.58. The fragments per kilobase million (FPKM) were used to evaluate gene expression levels by normalizing the length of genes using the count matrix. The R package limma^71^ was used to identify differentially expressed genes when the input was an FPKM matrix (Expression profiles retrieved from the GEO database).

### ChIP-Seq and data analysis

The ChIP-seq experiments were conducted as previously described^67^. The full length of *Gata2*-WT and *Gata2*-L359V were cloned into the MigR1-Flag plasmid vector and transfected into 32D cells. The anti-Flag antibody was used in the ChIP experiments. The ChIP-seq DNA libraries were constructed using the VAHTS Universal Pro DNA Library Prep Kit (Vazyme, Nanjing, China) according to the manufacturer’s instructions. The libraries were sequenced on the NovaSeq 6000. For ChIP-seq data analysis, all sequencing reads were mapped against the mouse genome (mm10) by using the bowtie2 (version 2.3.0)^72^ and uniquely mapped reads were kept for downstream analysis. The high confident binding peaks were called by overlapping peaks identified by the MACS suite (version 1.4.3)^73^ and the Homer Suite^74^. Visualization of the peaks was performed in the UCSC genome browser. The motif enrichment analysis was performed using the MEME suite (version 4.11.1)^75^. For GATA-switch analysis, the target genes of GATA1 and GATA2 were obtained from Fujiwara et al.^64^ (Table S6).

### Functional and pathway enrichment analysis of expression profiling data

GO enrichment analysis of differentially expressed genes between different groups was performed using both DAVID (https://david.ncifcrf.gov/) and STRING (https://string-db.org/) with the default parameters. Normalized RNA-seq data were rank-ordered by the fold change of gene expression between different groups. To identify the enriched pathways, GSEA^76^ was performed using R package clusterProfiler^77^. Gene sets enrolled in the study were downloaded from two databases, the Molecular Signatures Database (MSigDB) of the Broad Institute, and the Mammalian Phenotype (MP) Ontology^26^. HALLMARK gene sets (H) and MSigDB curated gene sets (C2, C5) were used to perform GSEA analysis^76^. Gene IDs transformation between human and mouse was performed using R package biomaRt (https://bioconductor.org/packages/biomaRt/). R package enrichplot (https://github.com/GuangchuangYu/enrichplot) was used to interpret enrichment results of GSEA. For integration analysis with other Gata2 hematopoietic mouse models, the gene expression profiling of *Gata2* KO and *Gata2* WT was collected from Gene Expression Omnibus (GEO) with accession id GSE133248^40^. Gene markers of murine hematopoietic cells (e.g., LT-HSC, ST-HSC, and MPP) were obtained from Haemopedia database (https://haemosphere.org/), which was published by Graaf et al.^78^.

### scRNA-seq and data analysis

GFP^+^ BM cells were harvested from leukemic *Gata2*^WT/L359V^-*BCR/ABL* and *Gata2*^WT/WT^-*BCR/ABL* mice and loaded onto a GemCode Single-Cell Instrument (10X Genomics, Pleasanton, CA, USA). Single-cell RNA-seq libraries were constructed using the Chromium Single-cell 3′ Library Kit (10X genomics) and analyzed on the Illumina NovaSeq 6000. The 10X Genomics cell ranger v2.1.1 was used for raw sequence alignment, filtering, barcode counting, and unique molecular identifier (UMI) counting. The gene-cell-barcode count matrix was analyzed using the R Seurat (v3.1.2) package^79^. Cells that expressed less than 500 genes or over 10% mitochondrial RNA were filtered out. Genes that expressed in less than 0.1% of total cells were removed. The normalization method was used to normalize the filtered gene expression count matrix using the default parameters and workflows provided by Seurat^79^. The expression data were merged and integrated using Seurat, and 2000 variable genes were identified for batch effect correction. A total of 4844 cells and 2943 cells were obtained from the *Gata2*^WT/WT^-*BCR/ABL* and *Gata2*^WT/L359V^-*BCR/ABL* mice, respectively. For visualization, tSNE was used^80^. Cell types in each cluster was identified referred to the top markers with adjusted *p*-value (p_val_adj) ≤ 0.05 and average log fold change (avg_logFC) ≥ 0.5. The lineages were identified by the top-expressed cluster-specific genes. The lineage-specific genes were selected according to the previous reports^54,55^.

### Statistical analysis

The Student unpaired two-tailed *t*-test was used for group comparisons. Differences were considered significant at *p* < 0.05. To decrease false-positive rates, we used FDR correction in multiple test analysis. R package ggplot2 and pheatmap were used for visualization. All statistical analyses were performed using the GraphPad Prism software (GraphPad Software, San Diego, CA) and R software (version 4.0.2, http://www.R-project.org).

### Data sharing statement

All sequencing data included in this study are available at Sequence Read Archive (SRA) database (PRJNA659109).

## Supplementary information

Supplementary Materials

Supplemental Table S1

Supplemental Table S2

Supplemental Table S3

Supplemental Table S4

Supplemental Table S5

Supplemental Table S6
