# Supplementary material for: Gata2-L359V impairs primitive and definitive hematopoiesis and blocks cell differentiation in murine chronic myelogenous leukemia model
Source: Cell Death Dis. 2021 Jun 2;12(6):568. doi: 10.1038/s41419-021-03826-1 (PMC8173010; doi:10.1038/s41419-021-03826-1)
Supplement: Supplementary file 1 — Supplementary Materials [file 41419_2021_3826_MOESM1_ESM.docx]

This file includes nine supplemental figures with legends.

**
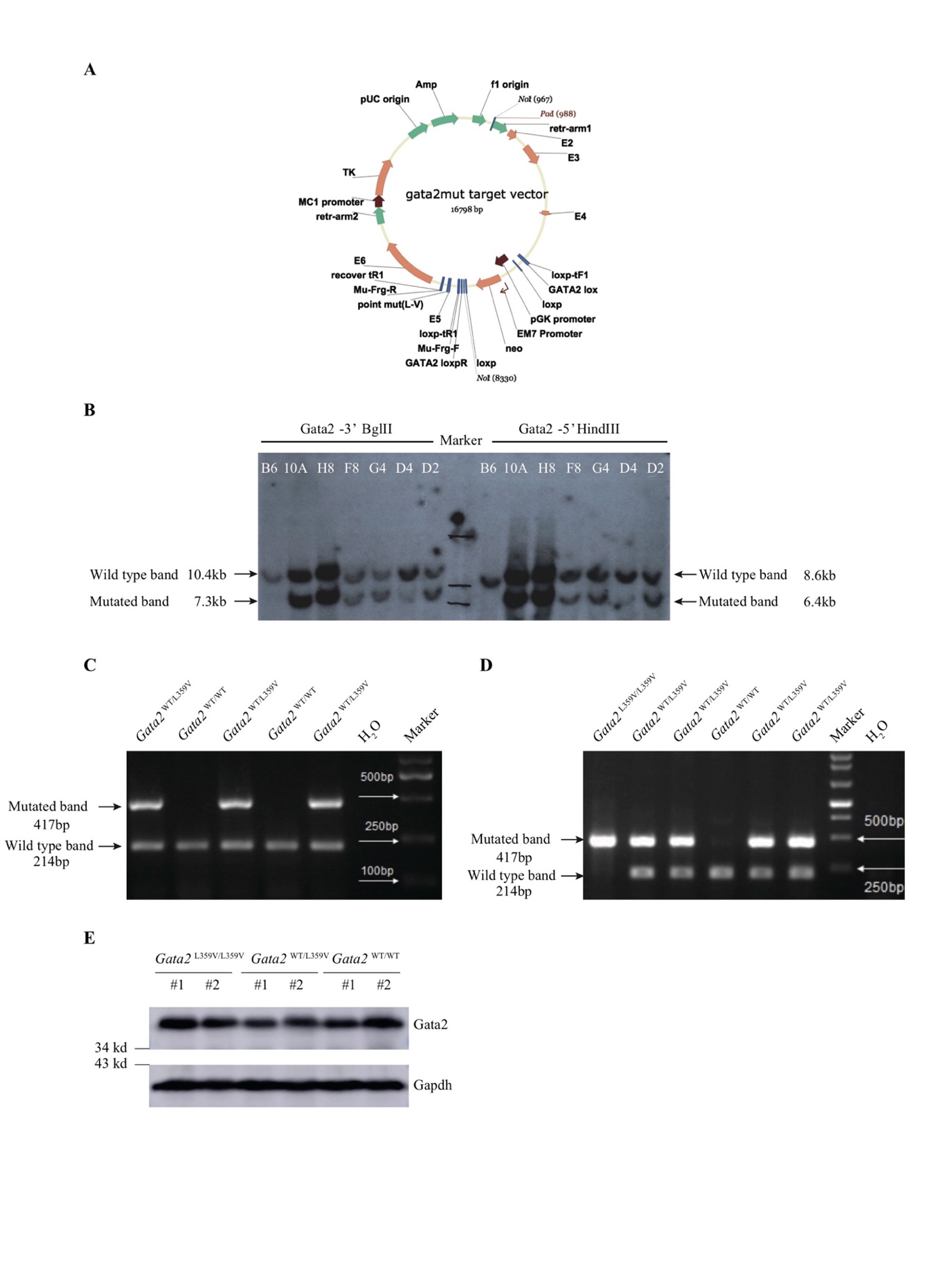
**

**Fig. S1: Construction of *Gata2*-L359V Knockin Mice.** (**A**) Schematic design of the *Gata2*-L359V knockin construct. (**B**) Southern blot analysis of targeted ES cells. (**C-D**) Representative illustration of PCR analysis for littermate (C) and embryo (D) genotyping. (**E**) Western blot analysis of Gata2 protein level in E10.5 embryos. Gata2 antibodies that can recognize both wild-type (WT) Gata2 and Gata2-L359V were used to detect the protein levels of Gata2-WT and Gata2-L359V.

**Fig. S2: Homozygous *Gata2*-L359V mutation impaired the embryonic hematopoiesis.** Statistical analysis of the cell counts of CD71^low^Ter119^low^, CD71^high^Ter119^low^, and CD71^high^Ter119^high^ cells between Gata2^WT/WT^ (n=3) and Gata2^L359V/L359V^ (n=3). Error bars represent the deviation from average. The statistical significance was indicated by *(P<0.05) and ** (P<0.01, Student’s *t-*test).

**Fig. S3: Transcriptome analysis of E9.5 yolk sacs.** (**A**) The expression analysis of embryonic erythropoiesis marker (*Cd71* and *Cd41*) and myeloid cell markers (*Kit*, *Cd45*, *Gfi1*, *Spi1*, and *Mpo*) in E9.5 yolk sac were plotted. (**B**) Homozygous *Gata2*-L359V mutation impaired the expression of genes essential for embryonic hematopoiesis. The expression levels of hemoglobin genes were validated by RT-qPCR in *Gata2*^WT/WT^ (n=9) and *Gata2*^L359V/L359V^ (n=6) yolk sacs. (**C**) Gene set enrichment analysis (GSEA) of differentially expressed genes in *Gata2*^WT/L359V^ and *Gata2*^L359V/L359V^ vs *Gata2*^WT/WT^ yolk sacs. HALLMARK gene sets and MSigDB curated gene sets (C2, C5) were used. The size of each circle represents the enriched gene ratios and the color represents the adjusted P values.


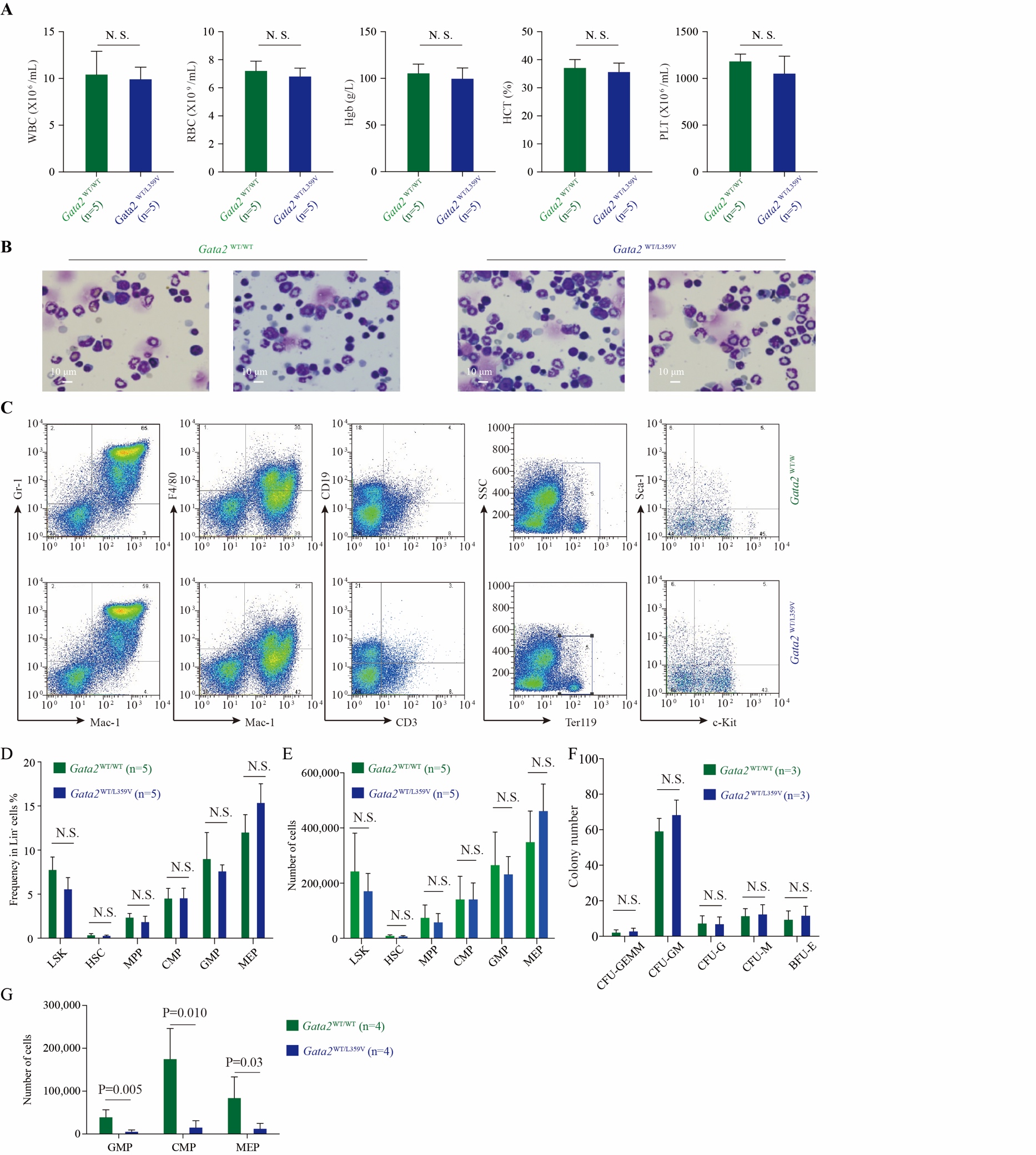


**Fig. S4: Phenotypic analysis of *Gata2*^WT/L359V^ and *Gata2*^WT/WT^ mice under steady-state conditions.** (**A**) Statistical analysis of routine blood test in the peripheral blood of 6-month-old mice. The levels of white blood cell counts (WBC), red blood cells (RBC), Hemoglobin (Hb), Hematocrit (HCT), and platelet (PLT) were plotted. (**B**) Representative morphology of bone marrow (BM) cells harvested from 6-month-old *Gata2*^WT/L359V^ and *Gata2*^WT/WT^ mice. (**C**) BM immunophenotype analysis of 6-month-old *Gata2*^WT/L359V^ and *Gata2*^WT/WT^ mice. No obvious differences were noted in terms of the percentage of cells in distinct compartments of the flow cytometry plots. (**D**) Statistical analysis of subsets of BM cells in 6-month-old *Gata2*^WT/L359V^ and *Gata2*^WT/WT^ mice. A combination of antibodies against the Sca1, Kit, CD150, and CD48 was used to identify distinct cell populations. The ratio of LSK (Lin^-^Sca1^+^c-Kit^+^), HSC (Hematopoietic stem cell, CD48^-^CD150^+^LSK), MPP (Multipotent progenitor, CD48^-^CD150^-^LSK), CMP (Common myeloid progenitor, Lin^-^Sca1^-^c-Kit^+^CD34^+^CD16/32^high^), GMP (Granulocyte-Macrophage progenitor, Lin^-^Sca1^-^c-Kit^+^CD34^+^CD16/32^low^) and MEP (Megakaryocyte-Erythrocyte, Lin^-^Sca1^-^c-Kit^+^CD34^-^CD16/32^-^) were plotted. (**E**) Quantification of cells in BM from Gata2^WT/WT^ and Gata2^WT/L359V^ mice. The cell numbers of LSK, HSC, MPP, CMP, GMP, and MEP were plotted. (**F**) Colony formation assay of the BM cells from 6-months-old *Gata2*^WT/L359V^ and *Gata2*^WT/WT^ mice. N. S.: No statistically significant difference. (**G**) Comparison of the number of donor-derived CMP (Lin^-^Sca1^-^c-Kit^+^CD34^+^CD16/32^high^), GMP (Lin^-^Sca1^-^c-Kit^+^CD34^+^CD16/32^low^) and MEP (Lin^-^Sca1^-^c-Kit^+^CD34^-^CD16/32^-^) in reconstituted BM cells between *Gata2*^WT/L359V^ (n=4) and *Gata2*^WT/WT^ (n=4) groups (Student’s *t-*test).


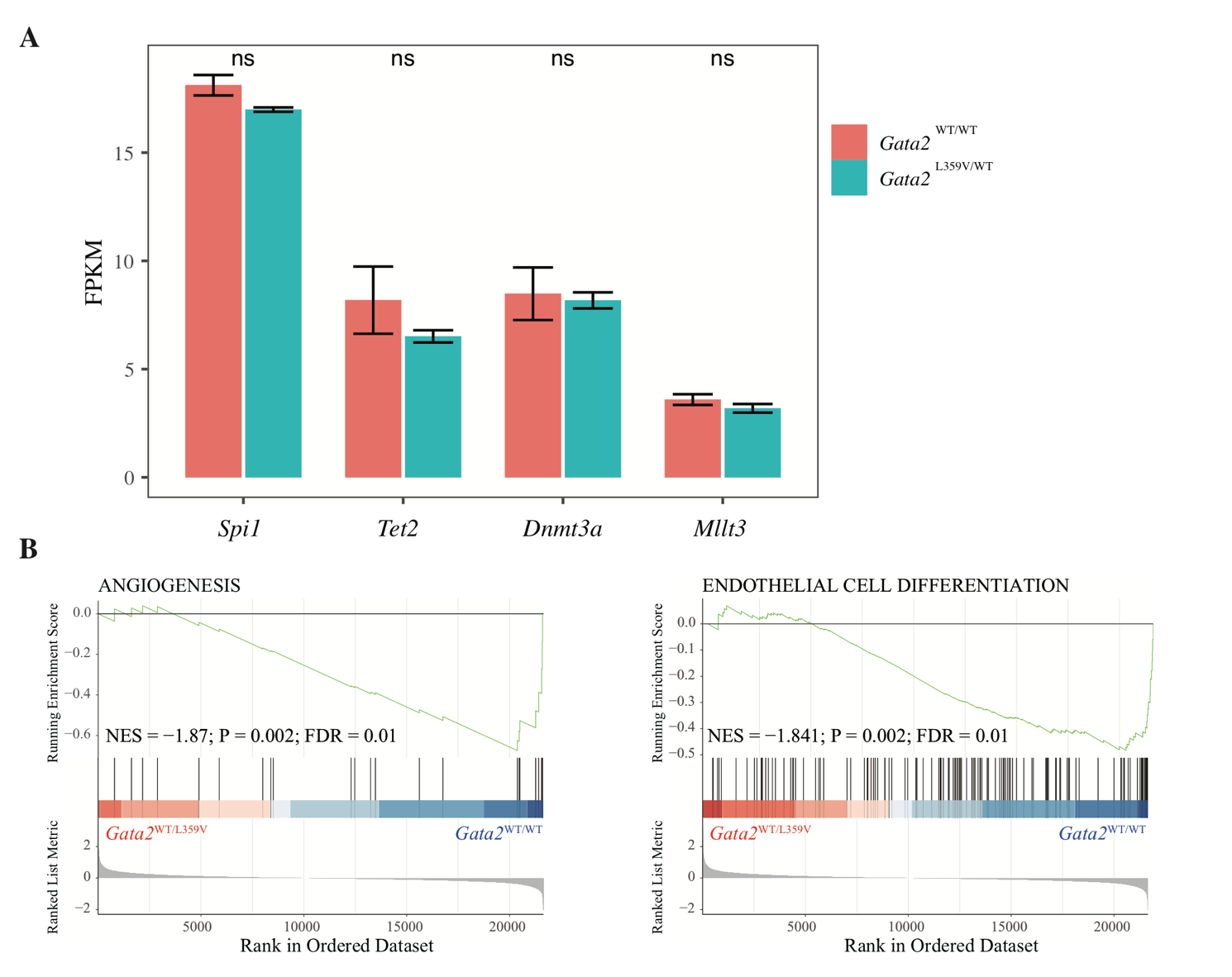


**Fig. S5: RNAseq analysis of LSKs in *Gata2*^WT/L359V^ versus *Gata2*^WT/WT^**. (A) Comparison of the expression levels of genes essential for HSC self-renewal in *Gata2*^WT/L359V^ and *Gata2*^WT/WT^ LSKs. The expression levels of *Spi1*, *Tet2*, *Dnmt3a*, and *Mllt3* in *Gata2*^WT/L359V^ and *Gata2*^WT/WT^ LSKs are plotted. (B) GSEA analysis of angiogenesis and endothelial cell differentiation pathways. Two gene sets (Genes related to angiogenesis and genes related to endothelial cell differentiation) that were reported to be down-regulated in *Gata2* over-expressed hematopoietic cells were used for GSEA analysis.

**
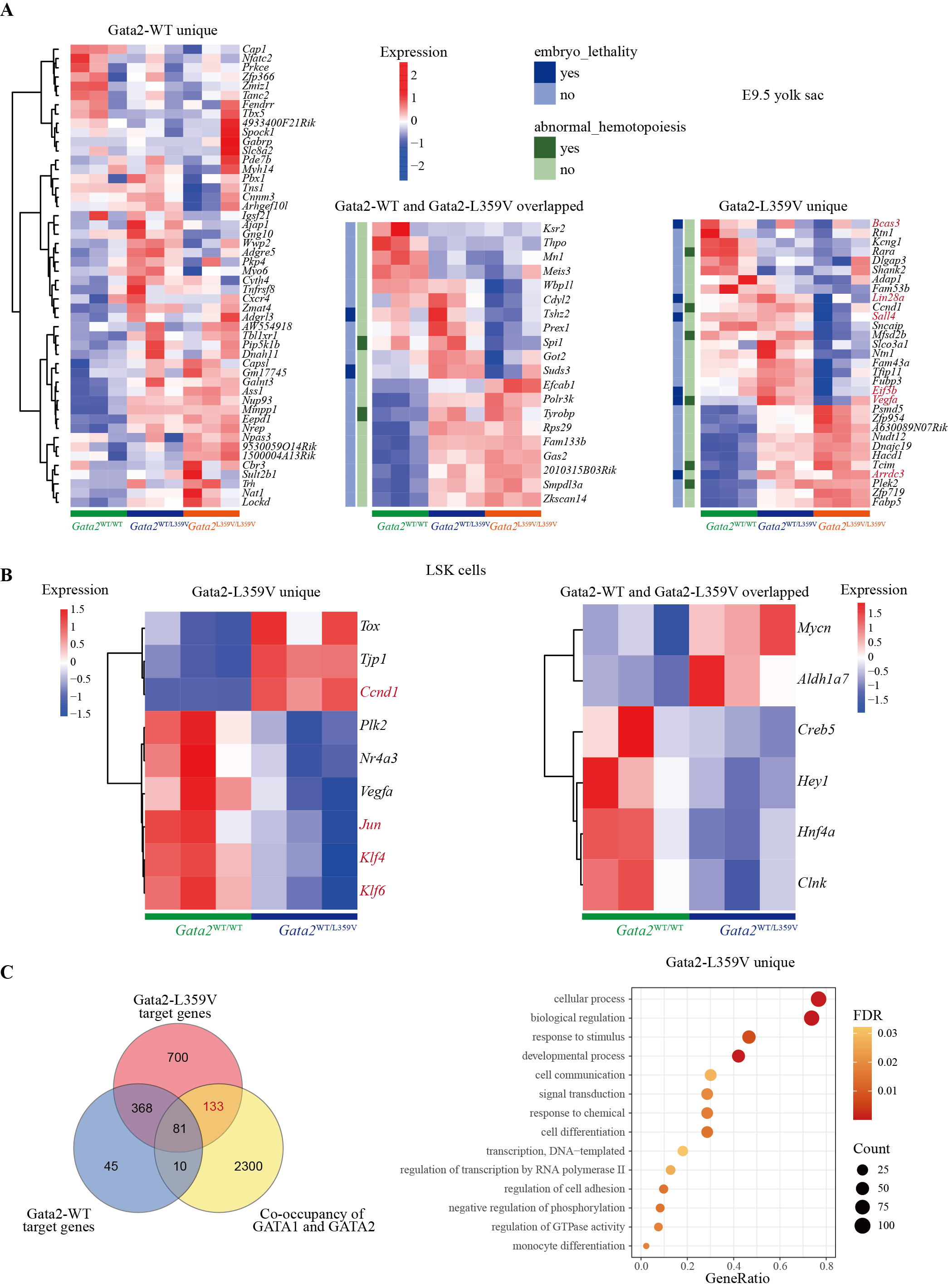
**

**Fig. S6: Combined analysis of ChIP-seq results in 32D cells with transcriptome data of yolk sacs and BM LSKs.** (**A**) Expression patterns of representative Gata2-WT unique, Gata2 overlapped, and Gata2-L359V unique genes in *Gata2*^WT/WT^, *Gata2*^WT/L359V^, and *Gata2*^L359V/L359V^ yolk sacs at E9.5. Relative expressions of genes in *Gata2*^WT/WT^, *Gata2*^WT/L359V^, and *Gata2*^L359V/L359V^ groups were illustrated by heatmap. Genes annotated to be associated with embryo lethality or abnormal hematopoiesis were also labeled with dark blue or dark green, respectively. (**B**) Expression patterns of representative Gata2-L359V unique and Gata2 overlapped genes in *Gata2*^WT/WT^ and *Gata2*^WT/L359V^ LSKs. (**C**) Overlap analysis between Gata2-L359V bound genes, Gata2-WT bound genes, and genes with co-occupancy of GATA1/GATA2 reported previously (left panel), followed by Gene ontology analysis (right panel). Targets identified by ChIP-seq were defined as Gata2-WT unique, Gata2-L359V unique, and Gata2 overlapped genes, respectively.


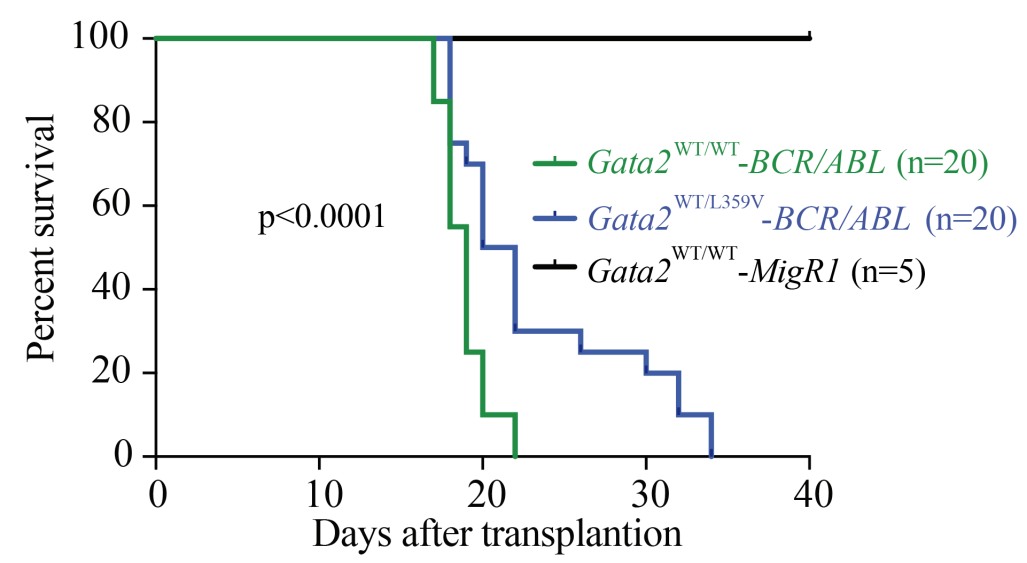


**Fig. S7: Survival analysis of *Gata2*^WT/L359V^*-BCR/ABL* and *Gata2*^WT/WT^*-BCR/ABL* mice.** *Gata2*^WT/WT^ and *Gata2*^WT/L359V^ BM cells were transduced with *BCR/ABL*-expressing retrovirus or the vehicle (MigR1). These cells were then injected into lethally irradiated recipients. Kaplan-Meier survival curves of *Gata2*^WT/L359V^-*BCR/ABL,* *Gata2*^WT/WT^-*BCR/ABL,* and *Gata2*^WT/WT^-MigR1 mice were shown.


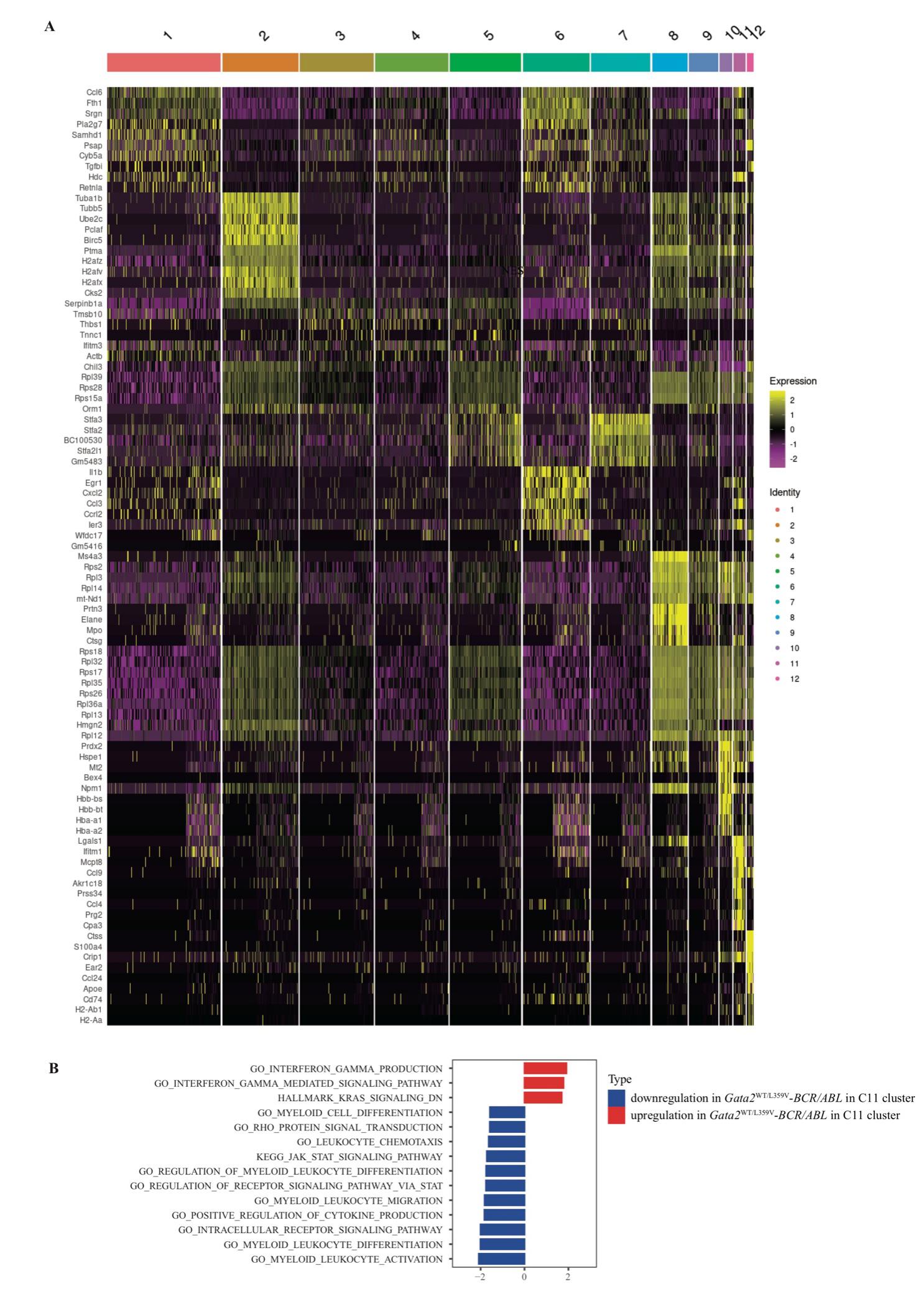


**Fig. S8: Single-cell RNA sequencing analysis of BM Cells from *Gata2*^WT/L359V^-*BCR/ABL* and *Gata2*^WT/WT^-*BCR/ABL* leukemic mice.** (**A**) Heatmap showing the expression patterns of representative genes in each cell cluster of GFP^+^ BM cells from *Gata2*^WT/L359V^-*BCR/ABL* and *Gata2*^WT/WT^-*BCR/ABL* leukemic mice. The yellow color indicates a high-level expression while the black and purple colors indicate low-level expression. (**B**) Summary of GSEA analysis of significantly differentially expressed genes between *Gata2*^WT/L359V^-*BCR/ABL* and *Gata2*^WT/WT^-*BCR/ABL* groups in C11 cluster.


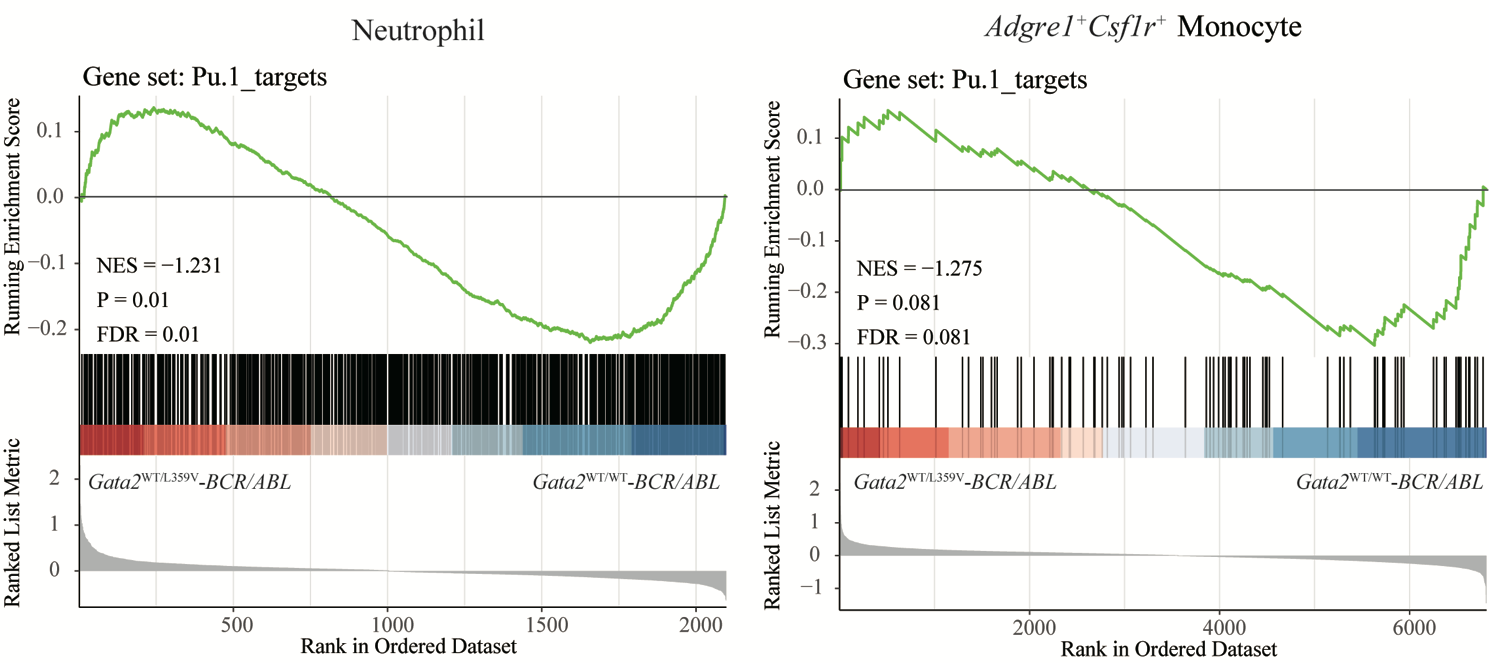


**Fig. S9: Impact of *Gata2*-L359V** **mutation** **on the expression of Pu.1 targets.** GSEA analyses of Pu.1_targets were conducted using the scRNA-seq data of neutrophil (cluster 1) and *Adgre1^+^Csf1r*^+^ monocytes (cluster 12). The gene rank list was generated by comparing the gene expression in Gata2^WT/L359V^-*BCR/ABL* vs Gata2^WT/WT^-*BCR/ABL* models.
