# Supplementary material for: Gata2-L359V impairs primitive and definitive hematopoiesis and blocks cell differentiation in murine chronic myelogenous leukemia model
Source: Cell Death Dis. 2021 Jun 2;12(6):568. doi: 10.1038/s41419-021-03826-1 (PMC8173010; doi:10.1038/s41419-021-03826-1)
Supplement: Supplementary file 2 — Supplemental Table S1 [file 41419_2021_3826_MOESM2_ESM.docx]

**Table S1. Time determination of *Gata2*-L359V transgenic embryonic death**

| **Stages** | **Number of pups** | **Gata2^WT/WT^** | | **Gata2^L359V/WT^** | | **Gata2^L359V/L359V^** | | |
| --- | --- | --- | --- | --- | --- | --- | --- | --- |
|  |  | **Alive** | **Dead** | **Alive** | **Dead** | **Alive** | **Dead** |  |
| **E9.5** | 98 | 23 | 0 | 49 | 0 | 26 | 0 |  |
| **E10.5** | 146 | 37 | 0 | 74 | 0 | 35 | 0 |  |
| **E11** | 42 | 13 | 0 | 20 | 0 | 9 | 0 |  |
| **E11.5** | 19 | 4 | 0 | 9 | 0 | 0 | 6 |  |
| **E12** | 15 | 4 | 0 | 7 | 0 | 0 | 4 |  |
| **E14** | 6 | 2 | 0 | 4 | 0 | 0 | 0 |  |
| **4 weeks** | 152 | 48 | 0 | 104 | 0 | 0 | 0 |  |
