# Supplementary material for: Gata2-L359V impairs primitive and definitive hematopoiesis and blocks cell differentiation in murine chronic myelogenous leukemia model
Source: Cell Death Dis. 2021 Jun 2;12(6):568. doi: 10.1038/s41419-021-03826-1 (PMC8173010; doi:10.1038/s41419-021-03826-1)
Supplement: Supplementary file 6 — Supplemental Table S5 [file 41419_2021_3826_MOESM6_ESM.docx]

**Table S5. Primers**

| **Hba-a1** | F:5’-CACCACCAAGACCTACTTTCC-3’  R:5’-CAGTGGCTCAGGAGCTTGA-3’ |
| --- | --- |
| **Hbb-y** | F:5’-TGGCCTGTGGAGTAAGGTCAA-3’  R:5’-GAAGCAGAGGACAAGTTCCCA-3’ |
| **Hba-x** | F:5’-CTACCCCCAGACGAAGACCTA-3’  R:5’-CTTAACCGCATCCCCTACGG-3’ |
| **Hbb-b1** | F:5’-GCACCTGACTGATGCTGAGAA-3’  R:5’-TTCATCGGCGTTCACCTTTCC-3’ |
| **Hbb-bh1** | F:5’-GAAACCCCCGGATTAGAGCC-3’  R:5’-GAGCAAAGGTCTCCTTGAGGT-3’ |
